# Marine Natural Products from the Beibu Gulf: Sources, Chemistry, and Bioactivities

**DOI:** 10.3390/md21020063

**Published:** 2023-01-19

**Authors:** Jiamin Wang, Yuning Qin, Miaoping Lin, Yingying Song, Humu Lu, Xinya Xu, Yonghong Liu, Xuefeng Zhou, Chenghai Gao, Xiaowei Luo

**Affiliations:** 1 Guangxi Key Laboratory of Marine Drugs, Institute of Marine Drugs, Guangxi University of Chinese Medicine, Nanning 530200, China; 2 CAS Key Laboratory of Tropical Marine Bio-Resources and Ecology/Guangdong Key Laboratory of Marine Materia Medica, South China Sea Institute of Oceanology, Chinese Academy of Sciences, Guangzhou 510301, China; 3 University of Chinese Academy of Sciences, 19 Yuquan Road, Beijing 100049, China

**Keywords:** marine natural product, the Beibu Gulf, chemical structures, bioactivity

## Abstract

Marine natural products (MNPs) play an important role in the discovery and development of new drugs. The Beibu Gulf of South China Sea harbors four representative marine ecosystems, including coral reefs, mangroves, seaweed beds, and coastal wetlands, which are rich in underexplored marine biological resources that produce a plethora of diversified MNPs. In our ongoing efforts to discover novel and biologically active MNPs from the Beibu Gulf, we provide a systematic overview of the sources, chemical structures, and bioactive properties of a total of 477 new MNPs derived from the Beibu Gulf, citing 133 references and covering the literature from the first report in November 2003 up to September 2022. These reviewed MNPs were structurally classified into polyketides (43%), terpenoids (40%), nitrogen-containing compounds (12%), and glucosides (5%), which mainly originated from microorganisms (52%) and macroorganisms (48%). Notably, they were predominantly found with cytotoxic, antibacterial, and anti-inflammatory activities. This review will shed light on these untapped Beibu Gulf-derived MNPs as promising lead compounds for the development of new drugs.

## 1. Introduction

Natural products have always been at the core of the development of organic chemistry, and as compound entity resources for the discovery of new drugs [1]. It is worth noting that unique marine environments provide a valuable platform for the discovery of drug compounds with novel structures exhibiting remarkable bioactivities [2]. Marine natural products possess obvious therapeutic effects on various human diseases, and they can resist viruses, tumors, inflammation, improve immune function, and prevent diabetes and Alzheimer’s disease. These factors have aroused great interest from chemists, biologists, and pharmacologists [3,4,5]. The first marine natural product with biological activity was officially reported by Bergman in the late 1950s [6]. Additionally, the isolation and investigation of MNPs is a rapidly expanding field of research at the interface of biology and chemistry. Looking back to 2009, when only 20,000 MNPs were known, the number has increased 50% in the past 11 years, which highlights the importance of marine habitats [7].

The Beibu Gulf is a semi-closed gulf located in the northwestern South China Sea, with Vietnam lying on the west of the gulf, and Guangxi Zhuang Autonomous Region and Hainan Island, both in China, lying to the north and east of the gulf, respectively. This Gulf is a global center of biodiversity and is an epicenter of marine biodiversity covering 12.8 million square kilometers of ocean and coastal waters. Additionally, this area harbors four representative marine ecosystems, including coral reefs, mangroves, seagrass beds, and coastal wetlands–rich in gas, oil, and biological resources–which exhibit an abundant biodiversity of marine micro-/organisms. To our knowledge, there are hitherto only a few reports about MNPs research in the Beibu Gulf. Therefore, these untapped and abundant biological resources will be of considerable interest to MNP chemists as well as marine biologists, in the coming decades [8,9,10]. 

Considering a plethora of underexplored marine biological resources inhabiting the Beibu Gulf, a systematic overview of the Beibu Gulf-derived MNPs has been carried out. We have performed a literature review of new chemical structures found in the Beibu Gulf. This review focuses on the comprehensive information from biological sources to pharmacological activities of a total of 477 MNPs derived from the Beibu Gulf, reported from November 2003 to September 2022. The sources, structures, and biological activities of these reviewed MNPs are discussed herein. We hope this review will shed light on the discovery of valuable MNPs, with potential therapeutic applications from the Beibu Gulf’s untapped biological resources.

## 2. New MNPs from the Beibu Gulf

### 2.1. Terpenoids

A total of 206 marine terpenoids were discovered within the period 2003–2022, consisting of 17 sesquiterpenoids, 76 diterpenoids, 42 triterpenoids and steroids, and 71 meroterpenoids. In addition, a total of 72 active compounds were found in marine terpenoids from the Beibu Gulf, including cytotoxic (24 compounds), anti-microbial (19 compounds), enzyme inhibitory (13 compounds), anti-inflammatory (10 compounds), anti-viral (5 compounds), and anti-fouling activities (2 compounds).

#### 2.1.1. Sesquiterpenoids

**Figure 1:**
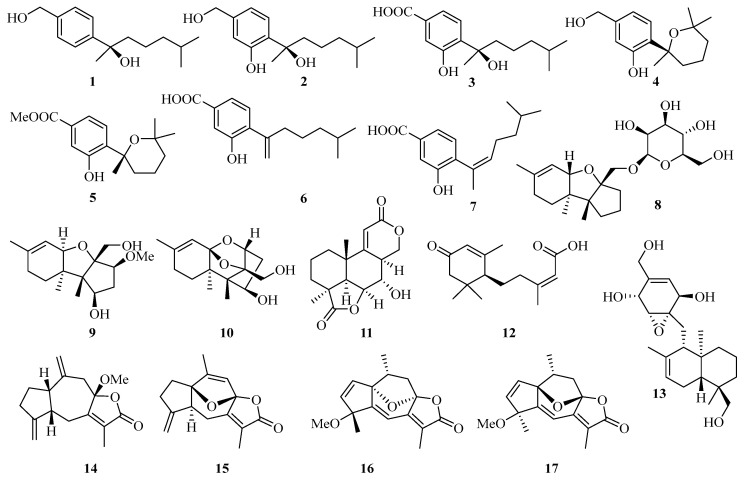
Chemical structures of sesquiterpenoids (**1**–**17**).

Four new bisabolane-type sesquiterpenoids, aspergiterpenoid A (**1**), (−)-sydonol (**2**), (−)-sydonic acid (**3**), and (−)-5-(hydroxymethyl)-2-(2′,6′,6′-trimethyltetrahydro-2Hpyran-2-yl) phenol (**4**) (Figure 1) were isolated from the fermentation broth of a marine-derived fungus *Aspergillus* sp., which was isolated from the sponge *Xestospongia testudinaria* collected from the Weizhou coral reef in the Beibu Gulf, Guangxi Autonomous Region, China. Compounds **1**−**4** showed selective antibacterial activities against eight bacterial strains with the minimum inhibiting concentrations (MIC) values between 1.3 and 20.0 µM [11]. 

Moreover, another three new phenolic bisabolane-type sesquiterpenoids: (+)-methyl sydowate (**5**), 7-deoxy-7,14-didehydrosydonic acid (**6**), and 7-deoxy-7,8-didehydrosydonic acid (**7**) were isolated from the fermentation broth of a marine-derived fungus *Aspergillus* sp., which was also obtained from the Weizhou coral reef in the Beibu Gulf of South China Sea [10]. 

Meanwhile, three new compounds of the trichothecene-based sesquiterpenes, chartarenes A–C (**8**–**10**), were isolated from the sponge (*Niphates recondite*)-associated fungus *Stachybotrys chartarum*. These compounds exert potent or selective inhibition against a panel of tumor cell lines including HCT-116, HepG2, BGC-823, NCI-H1650, and A2780, with IC_50_ values ranging from 0.01 to 10 μM [12]. 

Botryosphaerin F (**11**), a new sesquiterpene, was isolated from the mangrove fungus *Aspergillus terreus*, which showed a potent inhibiting activity towards MCF-7 and HL-60 cancer cell lines with 50% inhibition of cell growth (IC_50_) values of 4.5 and 3.4 μM, respectively [13]. An abscisic acid-type sesquiterpene, named (10*S*, 2*Z*)-3-methyl-5-(2,6,6-trimethyl-4-oxocyclohex-2-enyl) pent-2enoicacid (**12**), was isolated from mangrove endophytic fungus *Pleosporales* sp. SK7 [14]. 

Craterellin D (**13**), a new merosesquiterpenoid, was isolated from a soft coral-derived *Lophiostoma* sp. fungus, which was collected from the Weizhou Island coral reef [15]. A guaiane-type sesquiterpenoid (**14**) was obtained from gorgonian *Echinogorgia sassapo* reticulata collected from the Weizhou Islands in the Beibu Gulf [16]. Three new guaiane sesquiterpene lactones, echinofloranolides A–C (**15**–**17**) were isolated from the gorgonian *Echinogorgia flora* collected from Weizhou Island [17]. 

#### 2.1.2. Diterpenoids

**Figure 2:**
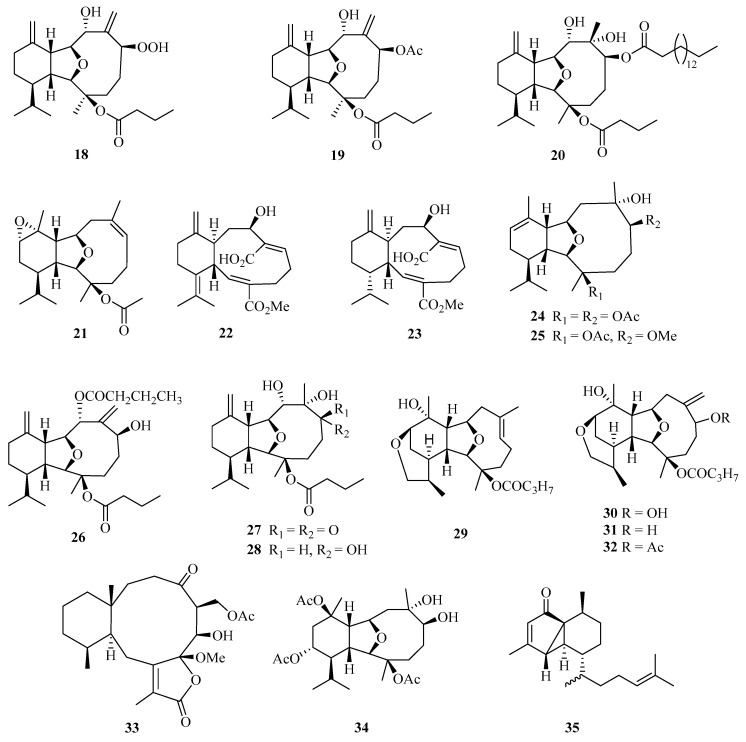
Chemical structures of diterpenoids (**18**–**35**).

A detailed chemical investigation of the Chinese soft coral *Cladiella krempfi* has yielded four new eunicellin-based diterpenoids, named oxylitophynol (**18**), litophynol A acetate (**19**), litophynol C (**20**), and krempfenin (**21**) (Figure 2). Particularly, compound **18** was shown to exhibit antiproliferative activity against C6 glioma cells, a multi-drug resistant tumor cell line, with an IC_50_ value of 21 ± 2 µM [18]. 

In addition, another two undescribed eunicellin diterpenoids, microeunicellols A (**22**) and B (**23**), were isolated from the culture of a bacterial symbiont, *Streptomyces albogriseolus* SY67903. Moreover, compound **22** displayed cytotoxicity against MCF-7 (IC_50_ = 5.3 μM) and MDA-MB-231 (IC_50_ = 8.6 μM) cell lines [19]. 

Bioactivity-guided isolation of the rare gorgonian *Muricella sibogae* yielded two new eunicellin-type diterpenes, sibogins A (**24**) and B (**25**). The cytotoxicity of **24** and **25** were evaluated in vitro against P388 and BEL-7402 cell lines. Both of them showed only weak activities against P388 cell lines, with inhibition rates ranging from 10 to 60% at a concentration of 50 mg/mL [20]. 

Meanwhile, three new polyoxygenated eunicellin-type diterpenes, namely, 8-n-butyryl-litophynol A (**26**), 6-keto-litophynol B (**27**), and 6-epi-litophynol B (**28**), were isolated from the soft coral *Cladiella krempfi* collected from Weizhou island. All the compounds exhibited moderate anti-inflammatory activities using inhibition of TNF-*α* with IC_50_ values of 15.8, 19.9, and 43.7 μM, respectively [21]. 

Four diterpenes, tritoniopsins A−D (**29**–**32**), have been isolated from Weizhou island; nudibranch *Tritoniopsis elegans* and its prey, the soft coral *Cladiella krempfi* [22]. Two new diterpenoids, anthogonoid A (**33**) and antsimplexin A (**34**), were isolated from a Beibu Gulf gorgonian coral, *Anthogorgia caerulea*. These compounds showed significant antifouling activity against the larval settlement of *Balanus amphitrite* with IC_50_ values of 5.3 and 2.9 μM, respectively [23]. Chemical examination of a Chinese gorgonian *Anthogorgia* sp. resulted in the isolation of one new compound, a rearranged serrulatane-type diterpenoid anthogorgiene P (**35**) [24]. 

**Figure 3:**
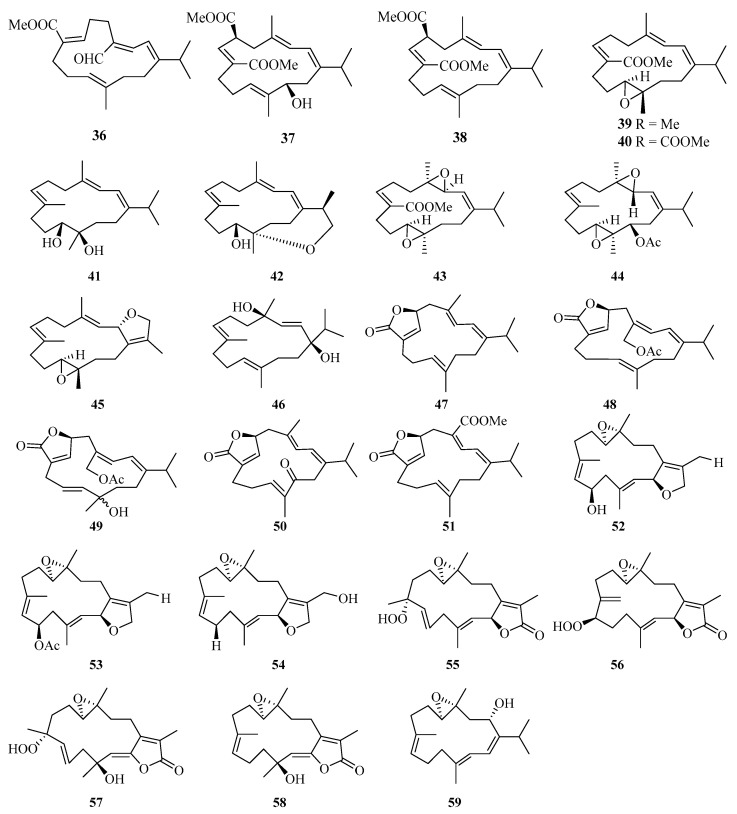
Chemical structures of diterpenoids (**36**–**59**).

Eleven new cembrane diterpenes, namely, sarcoehrenins A–J (**36**–**44**, **46**) and (2*S*,11*S*,12*S*)-isosarcophytoxide (**45**) (Figure 3), were isolated from the soft coral *Sarcophyton ehrenbergi* collected from the Weizhou Island [25]. A detailed chemical research of soft coral *Sarcophyton ehrenbergi* from Weizhou Island also yielded five new cembranoids (**47**–**51**). Notably, compound **48** exhibited a potent TNF-*α* inhibitory activity (IC_50_ = 8.5 μM), which was analogous to the positive control dexamethasone (IC_50_ = 8.7 μM) [26]. 

Moreover, another eight cembrane-type diterpenoids, namely, (+)-(6*R*)-6-hydroxyisosarcophytoxide (**52**), (+)-(6*R*)-6-acetoxyisosarcophytoxide (**53**), (+)-17-hydroxyisosarcophytoxide (**54**), sarcomililatins A–D (**55**–**58**), and sarcomililatol (**59**), were isolated from the soft coral *Sarcophyton mililatensis* collected from Weizhou Island. In addition, compound **55** showed a moderate inhibitory effect on the TNF*α*-induced nuclear factor kappa B (NF-*κ*B) activation, showing an IC_50_ value of 35.2 ± 12.4 μM [27]. 

**Figure 4:**
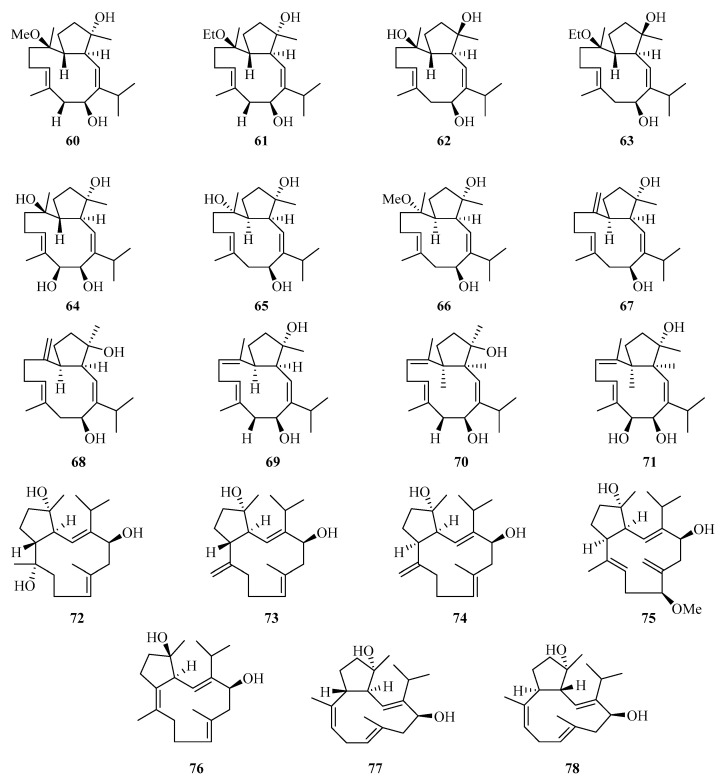
Chemical structures of diterpenoids (**60**–**78**).

Nineteen new cembranoids with unusual capnosane skeleton named trocheliophols A–S (**60**–**78**) (Figure 4) were isolated from the Chinese soft coral *Sarcophyton trocheliophorum*. Compounds **67**, **68**, and **78** exhibited potent inhibitory effects against phytopathogens and human disease-related Gram positive and negative bacteria, with the MIC values of 8.0–16.0 μg/mL [28].

Two new diterpenoids, hypoxyterpoids A (**79**) and B (**80**), were obtained from the crude extract of the mangrove-derived fungus *Hypoxylon* sp. Among them, compound **79** showed moderate *α*-glucosidase inhibitory activities with IC_50_ values of 74.2 ± 2.8 µM [29]. Two new brominated diterpenes, namely, laurendecumtriol (**81**) and 11-*O*-deacetylpinnaterpene C (**82**), were isolated and identified from the marine red alga *Laurencia decumbens* [30]. 

**Figure 5:**
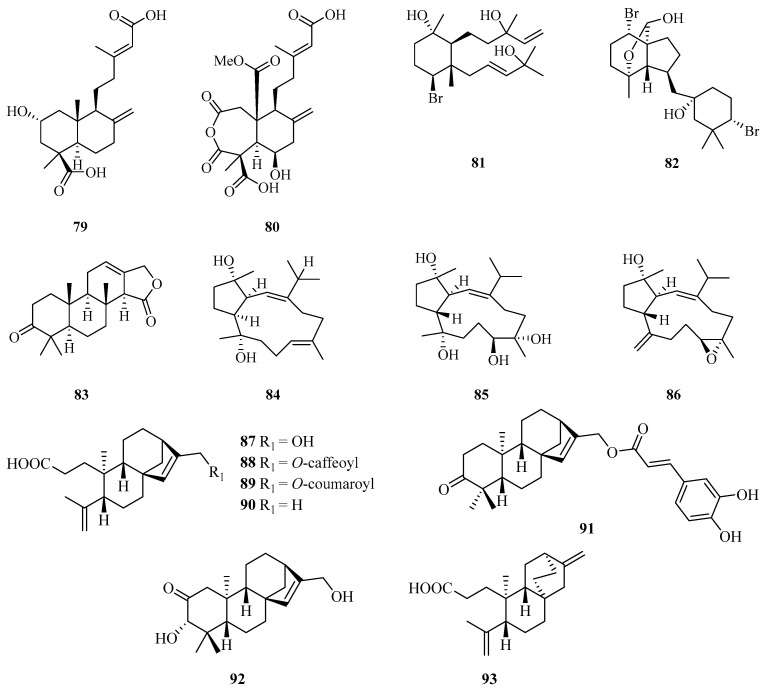
Chemical structures of diterpenoids (**79**–**93**).

A detailed chemical investigation of the nudibranch *Glossodoris atromarginata* collected from Weizhou Island, yielded a new spongian-type diterpene (**83**) (Figure 5) [31]. Three new diterpenoids with an unusual capnosane skeleton named lobophytrols A–C (**84**–**86**) were isolated from the soft coral *Lobophytum* sp. collected from Weizhou Island [32]. 

Chemical examination of the stems and twigs of the mangrove plant *Excoecaria agallocha* L. resulted in the isolation of six ent-kaurane diterpenoids named agallochaols K–P (**87**–**92**), and an atisane-type diterpenoid agallochaol Q (**93**). Compounds **87** and **91**–**93** showed anti-inflammatory potency to suppress expression of NF-*κ*B and AP1 targeted genes including TNF-*α* and IL-6 induced using lipopolysaccharide (LPS) in mouse macrophages Raw 264.7 cells, with inhibition rates exceeding 40% at the concentration of 1 *µ*g/mL. In addition, compounds **87** and **91**–**93** block NF-*κ*B activation, while **87** and **93** dramatically blocked AP-1 activation, indicating these compounds possess an anti-inflammatory potential in vitro [33]. 

#### 2.1.3. Triterpenoids and Steroids

**Figure 6:**
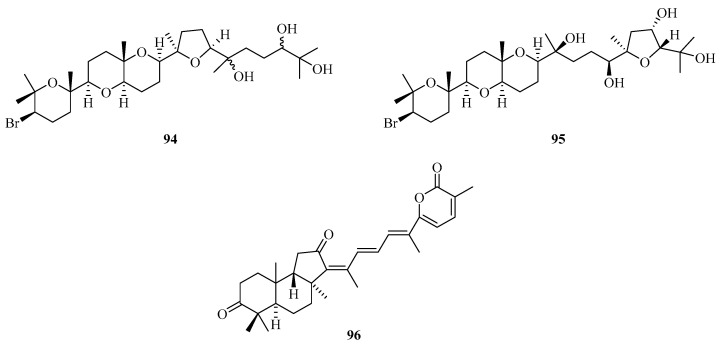
Chemical structures of triterpenoids (**94**–**96**).

Two new squalenoid-derived triterpenoids, namely, laurenmariannol (**94**) and (21a)21-hydroxythyrsiferol (**95**) (Figure 6), were isolated and identified from the marine red alga *Laurencia mariannensis*, which was collected off the coast of Hainan and Weizhou Islands. Compounds **94** and **95** displayed significant cytotoxic activities against P-388 tumor cells with IC_50_ values of 0.6 and 6.6 µg/mL, respectively [34]. One new triterpenoid (**96**) was isolated from sponge *Stelletta* sp., which was collected from Weizhou Island. [35]. 

**Figure 7:**
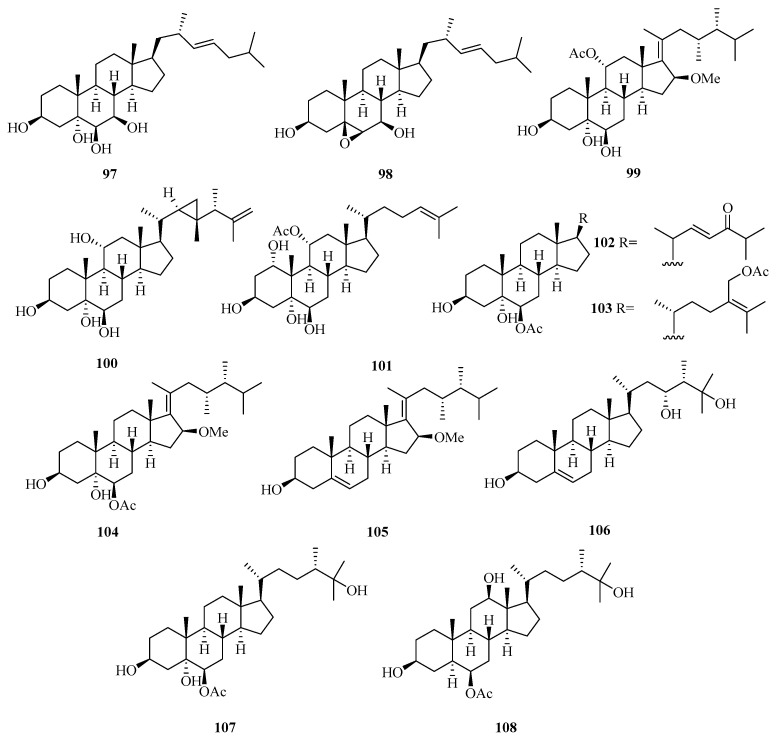
Chemical structures of steroids (**97**–**108**).

Two new polyhydroxylated sterols, named verumbsteroids A (**97**) and B (**98**) (Figure 7), were isolated from the gorgonian *Verrucella umbraculum* collected from Weizhou Island. Remarkably, compound **97** was found with cytotoxicity against five human tumor cell lines (HL-60, K562, HeLa, A-549, and HCT-116) with IC_50_ values ranging from 2.8 to 6.9 µM [36]. 

Chemical investigation on the soft coral *Sarcophyton* sp. collected from Weizhou Island yielded three new polyhydroxylated steroids, compounds (**99**–**101**). Compounds **100** and **101** exhibited potent activities against K562 cell lines with IC_50_ values ranging from 9.9 to 10.1 μM. Compound **99** potently inhibited the growth of HL-60 tumor cell lines, with an IC_50_ value of 9.3 μM [37]. 

Moreover, another four new polyhydroxylated steroids (**102**–**105**), were isolated from the soft coral *Sinularia acuta* collected from Weizhou Island. Compound **103** showed potent cytotoxicity against HL-60 and HeLa cell lines with IC_50_ values of 10.9 μM and 27.1 μM, respectively [38]. 

A chemical investigation of the ethanol extract of soft coral *Sinularia* sp. collected from Weizhou Island led to the isolation of three new polyoxygenated sterols, (3*S*,23*R*,24*S*)-ergost-5-ene-3*β*,23*α*,25-triol (**106**), (24*S*)-ergostane-6-acetate-3*β*,5*α*,6*β*,25-tetraol (**107**), and (24*S*)-ergostane-6-acetate-3*β*,6*β*,12*β*,25-tetraol (**108**) [39]. 

Meanwhile, another seven new polyoxygenated steroids (**109**–**115**) were isolated from the soft coral *Sarcophyton* sp. collected from Weizhou Island. In addition, all the metabolites exhibited antibacterial activity against the Gram-negative bacterium, *Escherichia coli*, and the Gram-positive bacterium, *Bacillus megaterium*, and antifungal activity against the fungi, *Microbotryum violaceum* and *Septoria tritici*, with MIC values ranging from 4.5 to 12.0 μg/mL [40]. 

**Figure 8:**
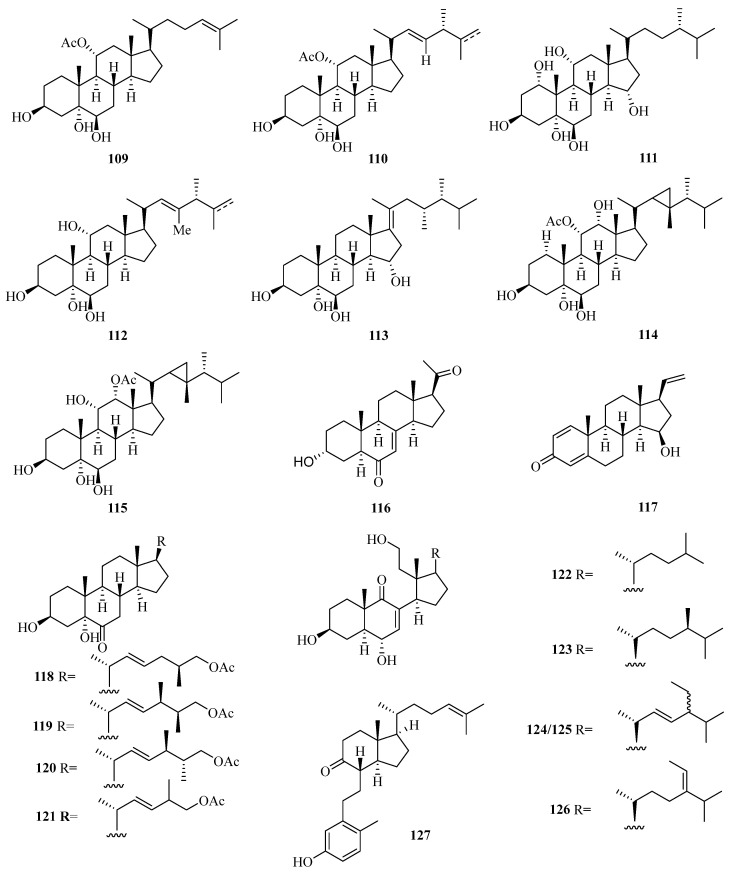
Chemical structures of steroids (**109**–**127**).

A new pregnane, 3*α*-hydroxy-7-ene-6,20-dione (**116**) (Figure 8), was obtained from the fungus *Cladosporium* sp. WZ-2008-0042 cultured from a gorgonian *Dichotella gemmacea,* collected from Weizhou Island, which exhibited potential activity against respiratory syncytial virus with the IC_50_ value of 0.1 mM [41]. Moreover, a new pregnane steroid (**117**) was isolated from a gorgonian *Carijoa* sp. collected from Weizhou Island, which exhibited cytotoxicity against the human hepatoma cell line Bel-7402, with IC_50_ value of 9.3 µM. Additionally, compound **117** exhibited promising antibacterial activity against *Pseudomona puido*, with an MIC value of 31.0 nM, which is approximately 5-fold more potent than ciprofloxacin (MIC = 156 nM) [42]. 

Four new steroids with an acetoxy linked at the end of the side chain, echrebsteroids A–D (**118**–**121**), were obtained from a gorgonian, *Echinogorgia rebekka*, found at Weizhou Island in the South China Sea. Remarkably, the (25*R*)-epimer (**120**) exhibited promising antiviral activity against the respiratory syncytial virus with an IC_50_ value of 0.2 μM and a comparatively higher therapeutic ratio (TC_50_/IC_50_ = 128) [43]. 

Five new 9,11-secosteroids (**122**–**126**), were isolated from a gorgonian, *Subergorgia suberosa*, found at Weizhou Island in the South China Sea. Among them, compounds **125/126** showed cytotoxic activities against the K562 cell line with the IC_50_ value of 8.1 μM [44]. One new 9,10-secosteroid, sibogol D (**127**), and two new 1,4-dien-3-one steroids, sibogols E (**128**) and F (**129**), were isolated from a gorgonian, *Muricella sibogae,* found at Weizhou Island in the South China Sea [45].

Bioactivity-guided isolation of the rare gorgonian *Muricella sibogae* yielded three new 9,10-secosteroids, sibogols A–C (**130**–**132**). The cytotoxicity of **130**–**132** were evaluated in vitro against the selected tumor cell lines P388 and BEL-7402. All the compounds showed only weak activities against P388 cell lines, with inhibition rates ranging from 10 to 60% at a concentration of 50 mg/mL [20]. 

**Figure 9:**
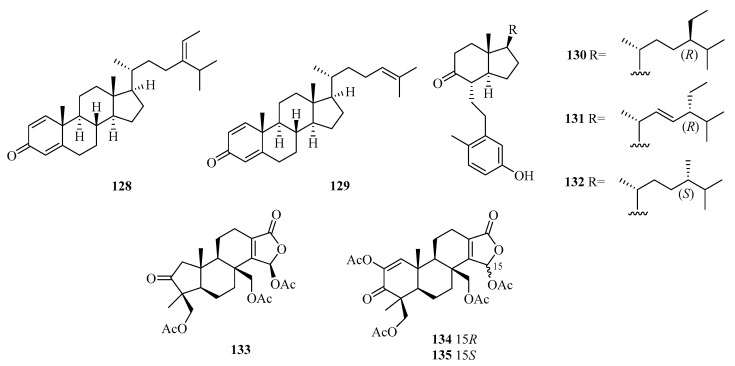
Structure of steroids (**128**–**135**) from Beibu Gulf.

Moreover, 3-Nor-spongiolide A (**133**), harboring the extremely rare 3-nor-spongian carbon skeleton, and spongiolides A (**134**) and B (**135**) (Figure 9), were isolated from the sponge *Spongia officinalis* collected from Weizhou Island [46]. 

#### 2.1.4. Meroterpenoids 

**Figure 10:**
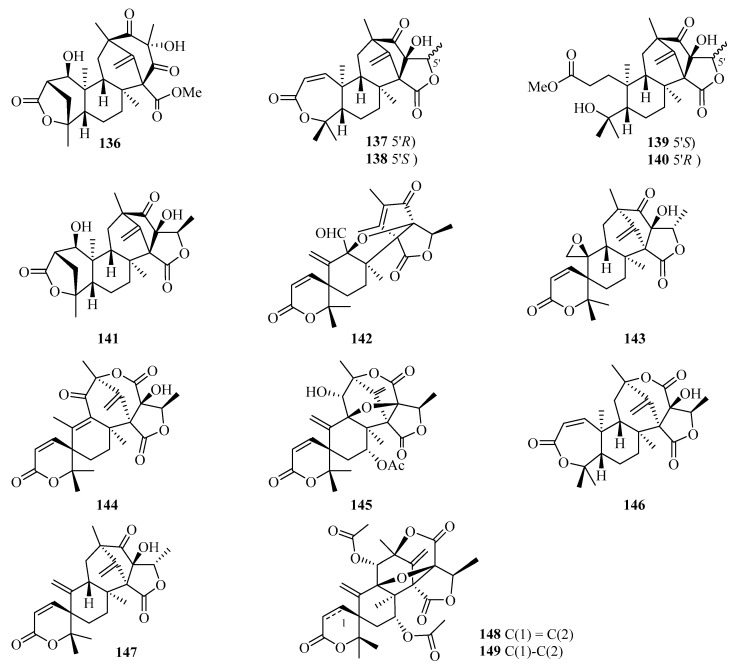
Chemical structures of meroterpenoids (**136**–**149**).

Chromatographic separation of the EtOAc extract of *Penicillium brasilianum* using a large-scale fermentation resulted in the isolation of twelve new 3,5-dimethylorsellinic acid-related meroterpenoids, namely brasilianoids A–L (**136**–**147**) (Figure 10). Among them, compound **136** significantly stimulated the expression of filaggrin and caspase-14 in HaCaT cells in a dose-dependent manner, while compounds **137** and **138** showed moderate inhibition against NO production in LPS-induced RAW 264.7 macrophages [47]. Moreover, only compound **137** exhibited significant inhibition against bacteria invasion into host cells, against the IEC-6 cells, with an IC_50_ value of 2.5 *µ*M [48]. Two new meroterpenoids, acetoxydehydroaustin B (**148**) and 1,2-dihydro-acetoxydehydroaustin B (**149**) were isolated in the form of a mixed crystal from the mangrove endophytic fungus *Aspergillus* sp. 085241B [49]. 

**Figure 11:**
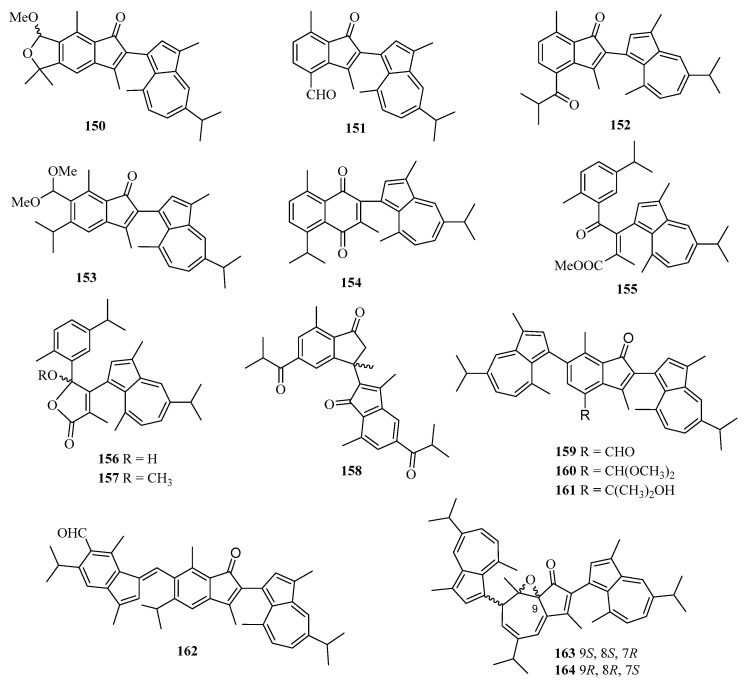
Chemical structures of meroterpenoids (**150**–**164**).

Chemical examination of a Chinese gorgonian *Anthogorgia* sp. resulted in the isolation of one new compound, a guaiazuene-based terpenoid, anthogorgiene Q (**150**) (Figure 11) [24]. Fourteen new guaiazulene-based terpenoids designated anthogorgienes A–O (**151**–**164**) were isolated from a Chinese gorgonian *Anthogorgia* sp. In particular, compound **157** showed selective inhibition against *Staphylococcus aureus* and *Staphylococcus pneumoniae* with IC_50_ values of 12.7–18.0 µM [50]. 

**Figure 12:**
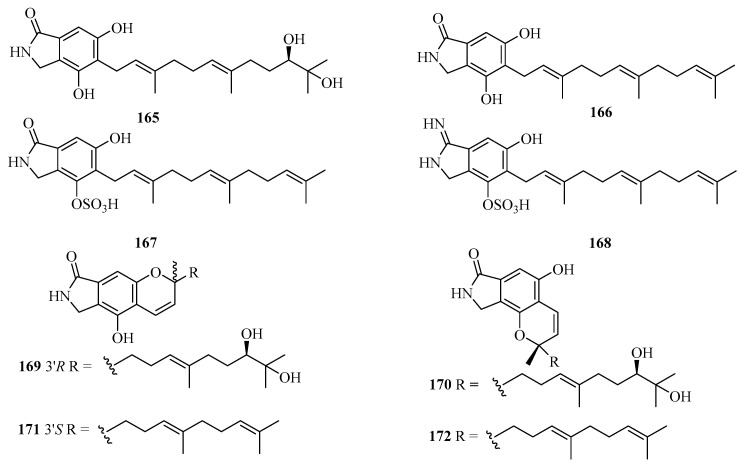
Chemical structures of meroterpenoids (**165**–**172**).

Eight new isoindolinone-type alkaloids, named chartarutines A–H (**165**–**172**) (Figure 12), were isolated from the sponge-associated fungus *Stachybotrys chartarum*. All compounds were evaluated for inhibition of the HIV-1 virus, while compounds **166**, **171**, and **172** exhibited significant inhibitory effects, with the IC_50_ values of 4.9, 5.6, and 5.6 μM, respectively [51]. 

**Figure 13:**
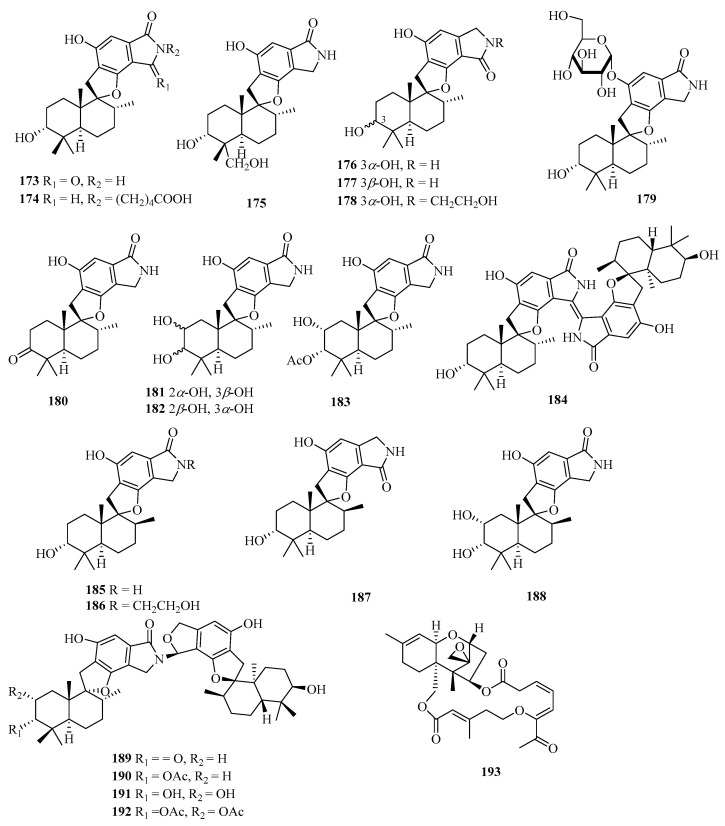
Chemical structures of meroterpenoids (**173**–**193**).

Chemical examination of the solid culture of the endophytic fungus *Stachybotrys chartarum* isolated from the sponge *Niphates recondita* resulted in the isolation of 16 new phenylspirodrimanes, named chartarlactams A–P (**173**–**188**) (Figure 13) [52]. Four new phenylspirodrimane-type dimers namely chartarlactams Q–T (**189**–**192**), were isolated from the fermentation-broth of a sponge-derived fungus *Stachybotrys chartarum* WGC-25C-6. Compounds **189**–**191** showed moderate inhibition against bacterial pathogen *Staphylococcus aureus* with MIC values ranging from 4 to 16 μg/mL, while compound **192** exhibited significant inhibition toward ZIKV virus [53]. Meanwhile, chartarene D (**193**), was isolated from the sponge (*Niphates recondite*)-associated fungus *Stachybotrys chartarum* [12]. 

**Figure 14:**
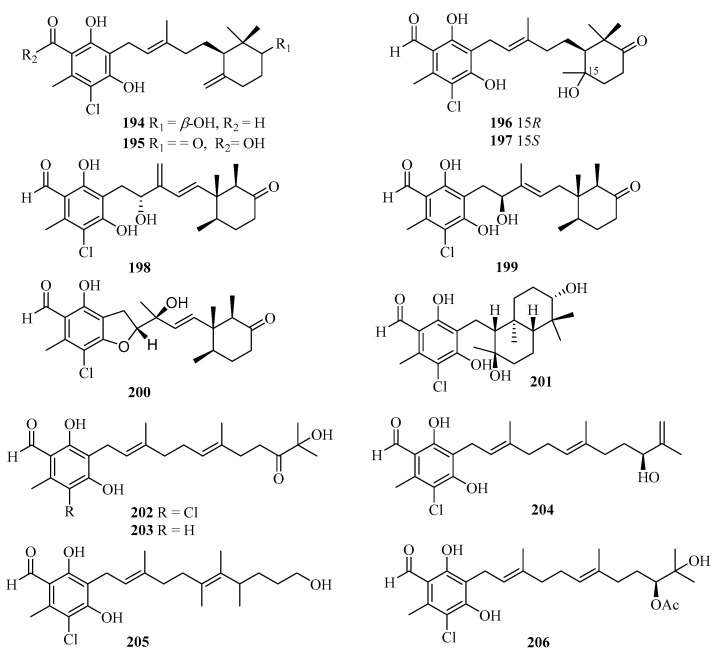
Chemical structures of meroterpenoids (**194**–**206**).

During our on-going studies aimed at discovering new secondary metabolites from the Beibu Gulf-derived marine fungi, 13 new ascochlorin derivatives, acremochlorins A–M (**194**–**206**) (Figure 14), were obtained from the coral-derived fungus *Acremonium sclerotigenum* by high-pressure liquid chromatography-diode array detector (HPLC-DAD)-guided isolation. Among them, compounds **194**, **198**, **199**, and **202**–**206** showed pronounced in vitro inhibitory activities against hDHODH with the IC_50_ values ranging from 0.07 to 9.3 μM [54]. Compounds **194** and **204**–**206** displayed antiproliferative activity against two triple-negative breast cancer (TNBC) cell lines, MDA-MB-231 and MDA-MB-468, with IC_50_ values of 0.48–12 μM. Notably, the novel and most potent hDHODH inhibitor (IC_50_ = 74 nM), acremochlorin A (**194**), also showed the strongest antiproliferative activity against MDA-MB-231 (IC_50_ = 0.65 μM) and MDA-MB-468 (IC_50_ = 0.48 μM), which was more potent than the positive controls cisplatin, 5-fluoro-2,4(1*H*, 3*H*)pyrimidinedione (5-Fu), and teriflunomide. Acremochlorin A (**194**) significantly inhibited TNBC cell growth and induced their apoptosis via hDHODH inhibition, and further efficiently suppressed tumor growth in patient-derived TNBC xenograft models without obvious body weight loss or overt toxicity in mice, highlighting a novel and potent hDHODH inhibitor as anti-TNBC therapeutic agents [54].

### 2.2. Polyketides

A total of 190 polyketides were discovered within the period, consisting of 43 fatty acids and linear molecules, 42 phenols, diphenyl ethers, and benzophenones, 32 benzofuranones, 32 quinones and xanthones, 11 macrolides, and 30 miscellaneous ones. In addition, a total of 73 active compounds were found among them, including anti-microbial (16 compounds), anti-inflammatory (16 compounds), enzyme inhibitory (15 compounds), cytotoxic (11 compounds), anti-fouling (7 compounds), anti-oxidative (6 compounds), and anti-viral activities (2 compounds).

#### 2.2.1. Fatty Acids and Linear Molecules

**Figure 15:**
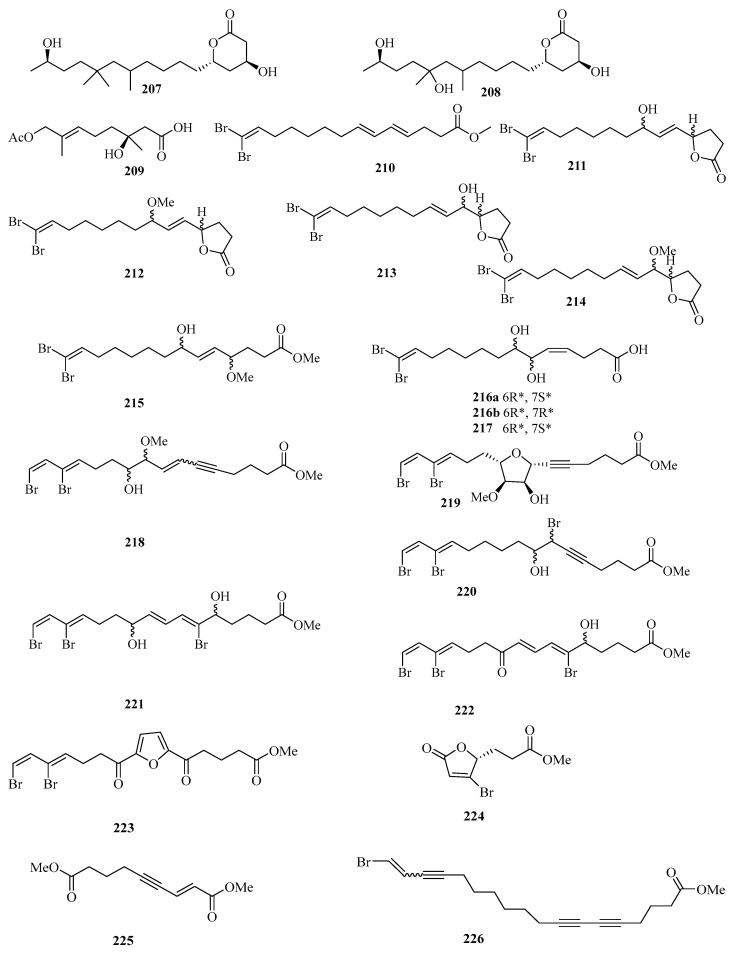
Chemical structures of fatty acids and linear molecules (**207**–**226**).

Chemical investigation of the fungal strain *Penicillium chrysogenum* QEN-24S resulted in the isolation of four new compounds. Their structures were identified as two polyketide derivatives penicitides A (**207**) and B (**208**) (Figure 15), and one monoterpene derivative penicimonoterpene (**209**). In addition, compound **209** displayed potent activity against the pathogen *Alternaria brassicae* with an inhibition zone of 17 mm in diameter at the concentration of 20 μg/disk, while compound **207** exhibited moderate cytotoxic activity against the human hepatocellular liver carcinoma cell line, with the IC_50_ value of 32 μg/mL [55]. 

A new brominated polyunsaturated lipid, methyl (*E*,*E*)-14,14-dibromo-4,6,13tetradecatrienoate (**210**), was isolated from the Et_2_O-soluble portion of the acetone extract of the Chinese marine sponge *Xestospongia testudinaria* treated with diazomethane [56]. Chemical examination of the Chinese marine sponge *Xestospongia testudinaria* led to the isolation of 16 new brominated polyunsaturated compounds (**211**–**226**), which were designated with the trivial names xestospongienes A–Z and Z1–Z13 [57]. 

**Figure 16:**
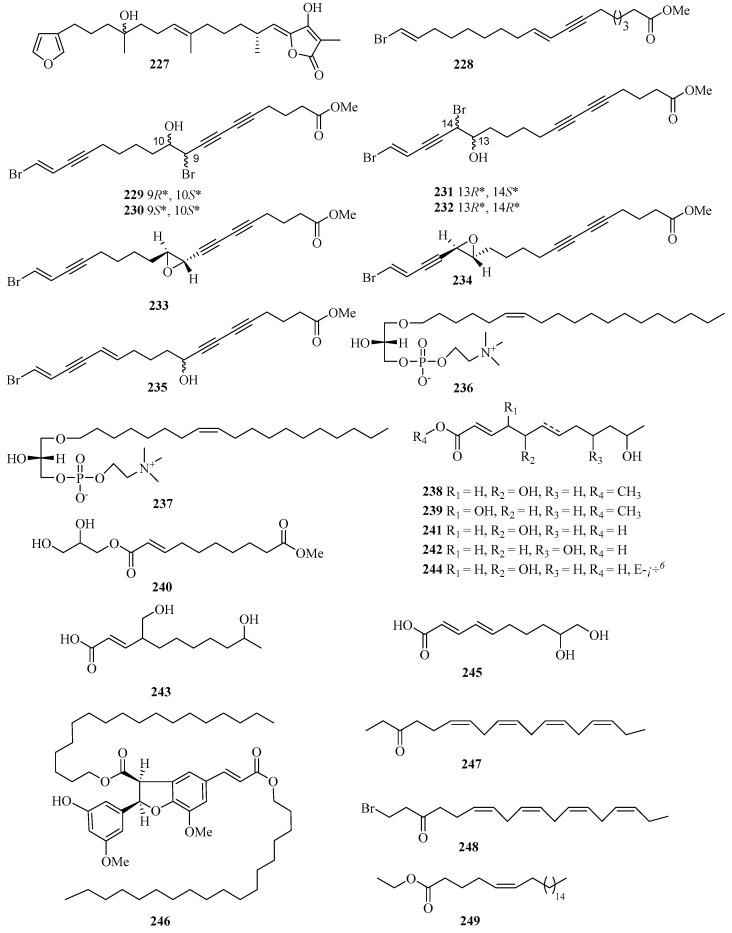
Chemical structures of fatty acids and linear molecules (**227**–**249**).

Chemical investigation of the acetone extract of the sponge *Biemna fortis* Topsent, collected from Weizhou Island, afforded a new minor furanosesterterpene, (12*E*,18*R*, 20*E*)-8-hydroxyvariabilin (**227**) (Figure 16) [58]. Chemical analysis of the Chinese marine sponge *Xestospongia testudinaria* afforded a library of brominated polyunsaturated lipids including eight new compounds, named xestonarienes A–H (**228**–**235**) [8]. 

Two new lysophospholipids (**236** and **237**) were isolated from the sponge *Spirastrella purpurea* from Weizhou Island, which displayed various moderate in vitro antifungal activities against the fungi *Cryptococcus neoformans*, with MIC values of 16 and 32 μg/mL, respectively [59]. Halotolerant fungus *Cladosporium cladosporioides* OUCMDZ-187 was isolated from the mangrove plant *Rhizophora stylosa* collected in Shankou, Guangxi Autonomous Region, China. Three new fatty acid esters cladosporesters A–C (**238**–**240**) and five new fatty acids cladosporacids A–E (**241**–**245**) were isolated from the ethyl acetate extract of the fermentation broth of *Cladosporium cladosporioides* OUCMDZ-187 in a hypersaline (10% salt) medium [60]. 

Brugnanin (**246**), a neolignan dioate, was isolated from a mangrove plant *Bruguiera gymnorrhiza*. MTT assay showed that **246** had weak inhibitory activity against the growth of CNE-1 nasopharyngeal carcinoma cell line, with the IC_50_ value of 57.2 μM [61]. Three new polyunsaturated lipids, (6*Z*,9*Z*,12*Z*,15*Z*)-octadeca-6,9,12,15-tetraen-3-one (**247**), (6*Z*,9*Z*,12*Z*,15*Z*)-1-bromooctadeca-6,9,12,15-tetraen-3-one (**248**), and (*Z*)-ethyl docos-5-enoate (**249**), were isolated from the marine sponge *Haliclona* sp., which was collected from Guangxi Autonomous Region, China, using HSCCC and HPLC methods [62]. 

#### 2.2.2. Phenols, Diphenyl Ethers, and Benzophenones

**Figure 17:**
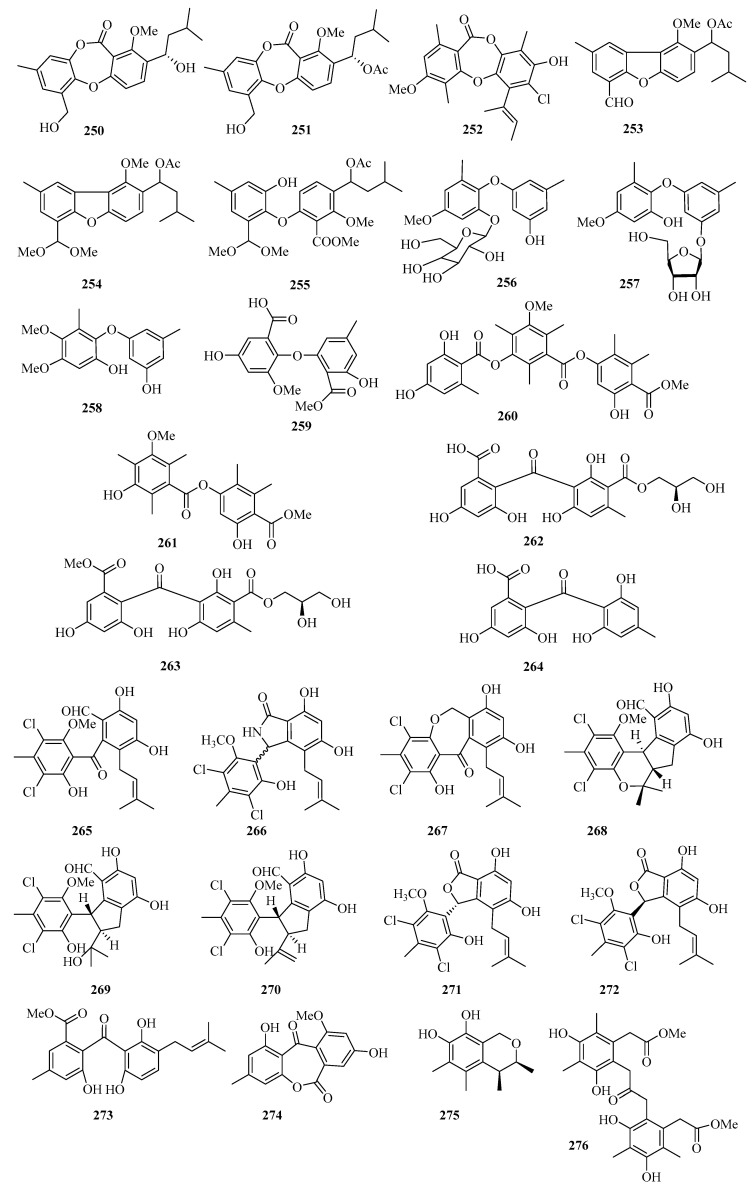
Chemical structures of phenols, diphenyl ethers, and benzophenones (**250**–**276**).

An endophytic fungus (*Talaromyces stipitatus* SK-4) was isolated from the leaves of a mangrove plant *Acanthus ilicifolius*. Its crude extract exhibited significant antibacterial activity and was purified to afford two new depsidones, talaromyones A (**250**) and B (**251**) (Figure 17). Remarkably, compound **251** showed antibacterial activity against *Bacillus subtilis* with an MIC value of 12.5 μg/mL. Compound **251** displayed moderate inhibitory activity against *α*-glucosidase with an IC_50_ value of 48.4 μM [63]. 

One new depsidone derivative, aspergillusidone H (**252**), was obtained from the Beibu Gulf coral-derived fungus *Aspergillus unguis* GXIMD 02505, which exhibited inhibition of LPS-induced NF-*κ*B in RAW 264.7 macrophages at 20 μM [64]. 

Three new diphenyl ether derivatives, talaromycins A–C (**253**–**255**), were isolated from a gorgonian-derived fungus, *Talaromyces* sp. In addition, compound **255** showed potent antifouling activity against the larval settlement of the barnacle *Balanus amphitrite* with the EC_50_ value of 2.8 μg/mL [65]. 

Three new diphenyl ether derivatives, phomaethers A–C (**256**–**258**), were isolated from a gorgonian-derived fungus *Phoma* sp. (TA07-1). Compounds **256** and **258** showed selective strong antibacterial activity against five pathogenic bacteria with MIC values and minimum bactericidal concentration (MBC) values between 0.2 and 10.0 μM [66]. Moreover, one new asterric acid derivative (**259**) was isolated from mangrove endophytic fungus *Pleosporales* sp. SK7 [14]. 

Two new polyketides, colletotrics B (**260**) and C (**261**), were isolated from the mangrove endophytic fungus *Phoma* sp. SYSU-SK-7. Compound **260** showed strong antimicrobial activity against the *Pseudomonas aeruginosa*, MRSA, and *Candida albicans* with MIC values in the range of 1.7–6.3 μg/mL. Furthermore, Compounds **260** and **261** also exhibited significant *α*-glucosidase inhibitory activities with IC_50_ values in the range of 36.2–90.6 μM [67]. 

Chromatographic separation of the bioactive extract resulted in the isolation of three new polyketide derivatives, eurobenzophenones A–C (**262**–**264**). Compound **264** exhibited potent radical scavenging activity against DPPH, with an IC_50_ value of 1.7 μM. Moreover, compound **263** (10 μM) exerted inhibitory activity against NO production in the LPS-induced BV2 cells of 17.4% [68]. 

A chemical investigation of the extract of the marine-sponge-associated fungus *Penicillium colombiensis* resulted in the isolation of eight chlorinated benzophenone derivatives, pestalone (**265**), pestalachlorides A–C, E, and F (**266**–**270**), pestalalactone (**271**), and pestalalactone atropisomer (**272**) [69]. One new prenylated polyketide, ascomarugosin A (**273**) was obtained from the culture of a mangrove endophytic fungus *Ascomycota* sp. SK2YWS-L [70]. 

A new lactone, 1,8-dihydroxy-10-methoxy-3-methyldibenzo[b,e]oxepine-6,11-dione (**274**), was isolated from a mangrove endophytic fungus *Phoma* sp. SK3RW1M collected from Weizhou Island [71]. Two undescribed compounds, guhypoxylonols C (**275**), and D (**276**), were isolated from the mangrove endophytic fungus *Aspergillus* sp. GXNU-Y45, which showed inhibitory activities against the production of NO, with IC_50_ values of 18.0 ± 0.1 and 16.7 ± 0.2 μM, respectively [72]. 

**Figure 18:**
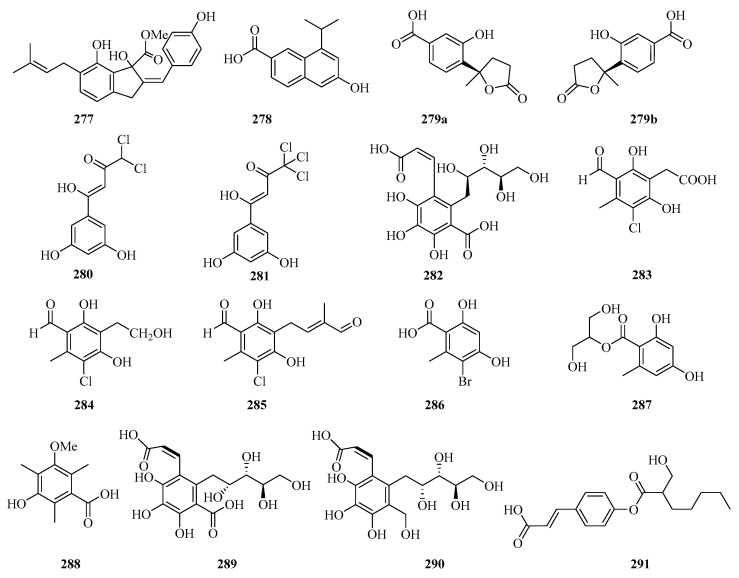
Chemical structures of phenols, diphenyl ethers, and benzophenones (**277**–**291**).

A new indene derivative named phomoindene A (**277**) (Figure 18) was isolated from the marine fungus *Phomopsis* sp. (No. GX7-4A). Preliminary pharmacological tests revealed that its IC_50_ values of cytotoxic activity against KB, KBv200, and MCF-7 cell lines were above 50 μM [73]. Leptosnaphthoic acid A (**278**), was isolated from an endophytic fungus *Leptosphaerulina* sp. SKS032, which exhibited antibacterial activity against *Staphylococcus aureus* with an MIC value of 50.0 μg/mL [74]. 

A pair of norbisabolane enantiomers: (+)-1-hydroxyboivinianic acid (**279a**), (−)-1-hydroxyboivinianic acid (**279b**), were obtained from the mangrove endophytic fungus *Aspergillus versicolor* SYSU-SKS025. Both of them showed moderate inhibitory activities against *α*-glucosidase with IC_50_ values of 60.1 and 120.3 μM, respectively [75]. From the oligotrophic culture of a soft coral-associated actinomycetes strain, *Streptomyces* sp. OUCMDZ-1703, was isolated and identified two new chlorinated phenols, strepchloritides A (**280**) and B (**281**). Both of them displayed cytotoxicity against MCF-7 cells with IC_50_ values of 9.9 and 20.2 μM, respectively [76]. 

Sonneradon A (**282**), isolated from the edible fruits of mangrove *Sonneratia apetala*, showed remarkable antiaging activity, which inhibited production of reactive oxygen species (ROS) by 53%, and reduced the accumulation of aging markers such as lipids and lipofuscins [77]. Three new chlorinated orsellinic aldehyde derivatives, orsaldechlorins A–C (**283**–**285**) and a naturally new brominated orsellinic acid (**286**), were identified from the Beibu Gulf coral-derived fungus *Acremonium sclerotigenum* GXIMD 02501. Compounds **283** and **284** showed inhibition of LPS-induced NF-*κ*B activation in RAW 264.7 macrophages at 20 μM. Moreover, the two new potent inhibitors (**283** and **284**) suppressed RANKL-induced osteoclast differentiation without cytotoxicity in bone marrow macrophage cells [78]. Chemical investigation of the fungal strain *Penicillium chrysogenum* QEN-24S resulted in the isolation of one glycerol derivative, 2-(2,4-dihydroxy-6-methylbenzoyl)-glycerol (**287**) [55]. 

One new polyketide, 3-hydroxy-5-methoxy-2,4,6-trimethylbenzoic acid (**288**), was isolated from the mangrove endophytic fungus *Phoma* sp. SYSU-SK-7, which showed strong antimicrobial activity against the *Bacillus subtilis* and *Candida albicans* with MIC values of 5.3 and 2.6 μg/mL, respectively. Furthermore, Compound **288** also exhibited weak *α*-glucosidase inhibitory activity with an IC_50_ value of 53.3 μM [67]. 

The chemical investigation of the mangrove fruit *Sonneratia apetala,* collected from the Beibu Gulf, led to the isolation of three new compounds, sonneradons A–C (**289**–**291**). Furthermore, compound **289** showed most significant antiaging effects on *Caenorhabditis elegans*, increasing the survival time of *Caenorhabditis elegans* from 30.8 ± 0.7% at the concentration of 100 μM to 34.5 ± 0.9% at the concentration of 300 μM, respectively. Moreover, compound **289** can significantly attenuate the aging-related decrease of pumping and bending in the worms of the healthspan essay, which suggests that compound **289** possesses great potential in antiaging applications [79]. 

#### 2.2.3. Benzofuranones

**Figure 19:**
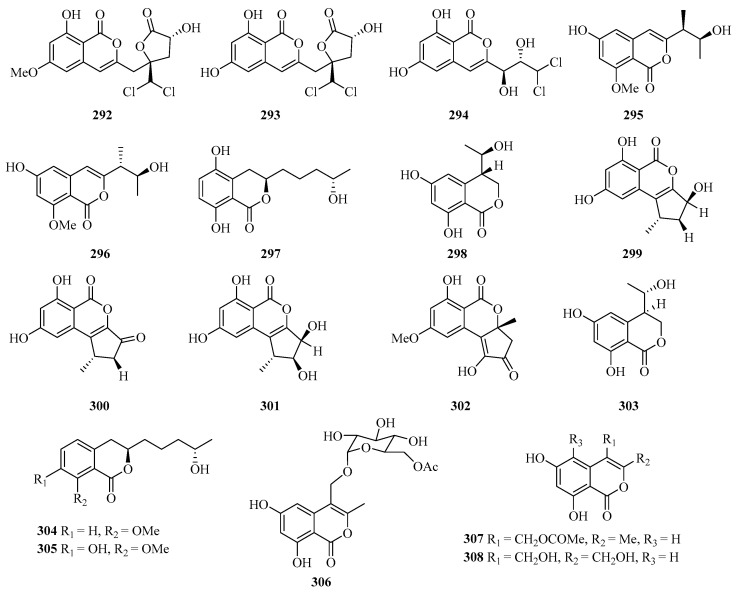
Chemical structures of benzofuranones (**292**–**308**).

Three new isocoumarins, dichlorodiaportintone (**292**), desmethyldichlorodiaportintone (**293**), and desmethyldichlorodiaportinol (**294**) (Figure 19), were isolated from the culture of the mangrove endophytic fungus *Ascomycota* sp. CYSK-4 from *Pluchea indica*. In particular, compound **293** showed significant anti-inflammatory activity through inhibiting the production of NO in LPS-induced RAW 264.7 cells with an IC_50_ value of 15.8 μM, while compound **292** exhibited weak activity with an IC_50_ value of 41.5 μM. In addition, compound **292** showed an antibacterial effect against *Staphylococcus aureus*, *Bacillus subtilis*, *Escherichia coli*, *Klebsiella pneumoniae*, and *Acinetobacter calcoaceticus* with the MIC values in the range of 25–50 μg/mL [80]. 

Meanwhile, another four new isocoumarin derivatives, hypoxymarins A–D (**295**–**298**), were obtained from the crude extract of the mangrove-derived fungus *Hypoxylon* sp. Particularly, compound **297** exhibited DPPH-scavenging activity with an IC_50_ value of 15.4 ± 0.2 μM [29]. 

Four new altenusin derivatives (**299**–**302**) were isolated from a culture of the endophytic fungus *Alternaria* sp. SK6YW3L, which was isolated from a fresh fruit of the mangrove plant *Sonneratia caseolaris*, collected from Weizhou Island. Compounds **300** and **301** exhibited moderate *α*-glucosidase inhibitory activities, with IC_50_ values of 78.2 and 78.1 μM, respectively [81]. 

Six new fungal metabolites, including three hydroisocoumarins, penicimarins A–C (**303**–**305**) and three isocoumarins, penicimarins D–F (**306**–**308**), were obtained from the sponge-derived fungus *Penicillium* sp. MWZ14-4, collected from the Weizhou Island. Among them, compound **308** exhibited inhibitory activity against *Staphylococcus aureus* with the MIC value of 12.5 μM [82]. 

**Figure 20:**
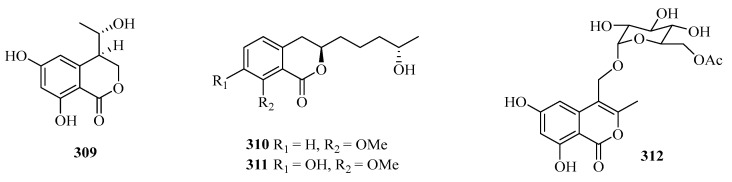

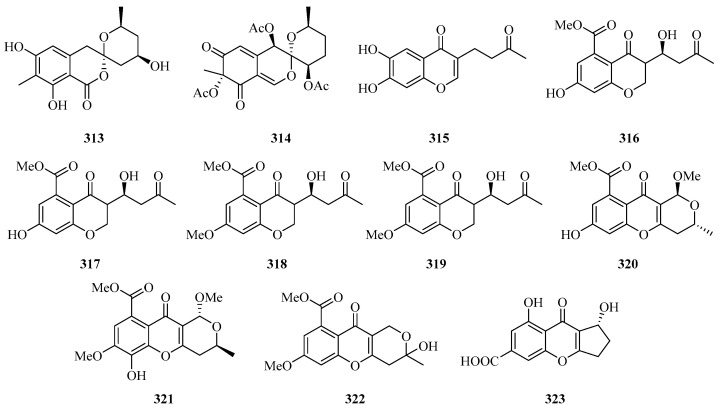
Chemical structures of benzofuranones (**309**–**323**).

Four benzofurans, penicifurans A–D (**309**–**312**) (Figure 20), were obtained from the sponge-derived fungus *Penicillium* sp. MWZ14-4, collected from the Weizhou Island. Among them, compound **309** exhibited inhibitory activity against *Staphylococcus albus* with a MIC value of 3.1 μM [82]. Two new azaphilone compounds, daldinins G (**313**) and H (**314**), were isolated from the soft coral-derived fungus *Penicillium glabrum* glmu003 [83]. 

One new polyketide, chaetochromone D (**315**), was isolated from the mangrove endophytic fungus *Phoma* sp. SYSU-SK-7. Furthermore, Compound **315** also exhibited significant *α*-glucosidase inhibitory activity with an IC_50_ value of 90.6 μM [67]. 

Seven new compounds: diaporchromanones A–D (**316**–**319**), (−)-phomopsichin A (**320**), (+)-phomopsichin B (**321**), and (−)-diaporchromone A (**322**), were isolated from an endophytic fungus *Diaporthe phaseolorum* SKS019. Compounds **318**–**321** exhibited moderate inhibitory effects on osteoclastogenesis by suppressing the receptor activator of NF-*κ*B with ligand-induced NF-*κ*B activation, with IC_50_ values from 28 to 46 μM [84]. 

An uncommon carboxyl group at C-8, coniochaetone K (**323**), was obtained from the Beibu Gulf-derived coral symbiotic fungus *Cladosporium halotolerans* GXIMD 02502. The in vitro cytotoxicity of **323** against two human prostatic cancer cell lines, C4-2B and 22RV1, was evaluated. It demonstrated significant cytotoxicity with inhibition of 64.6% at 10 μM [85].

#### 2.2.4. Quinones and Xanthones

**Figure 21:**
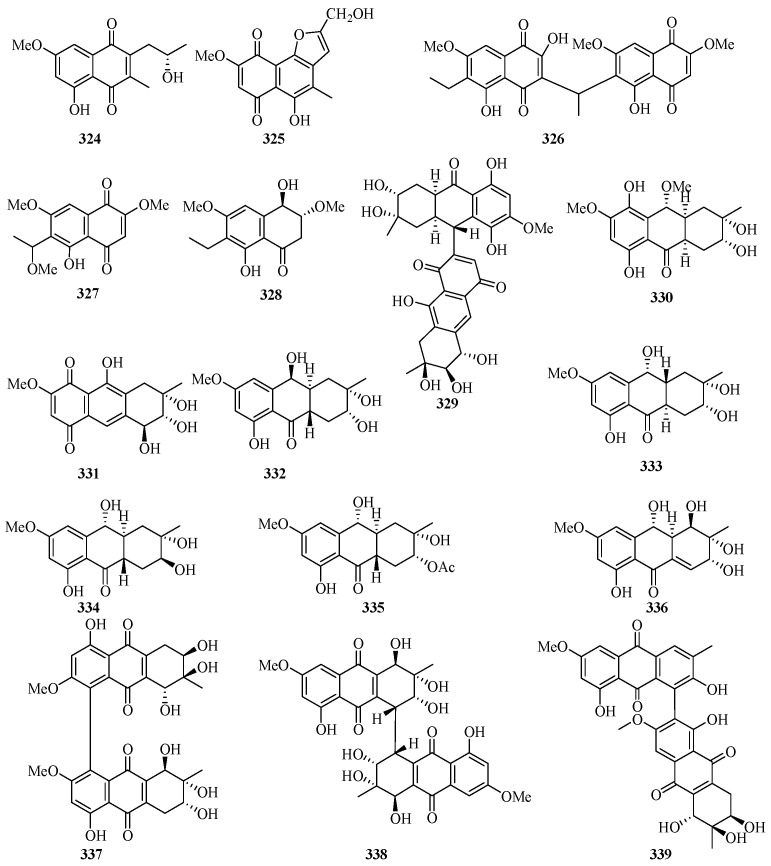

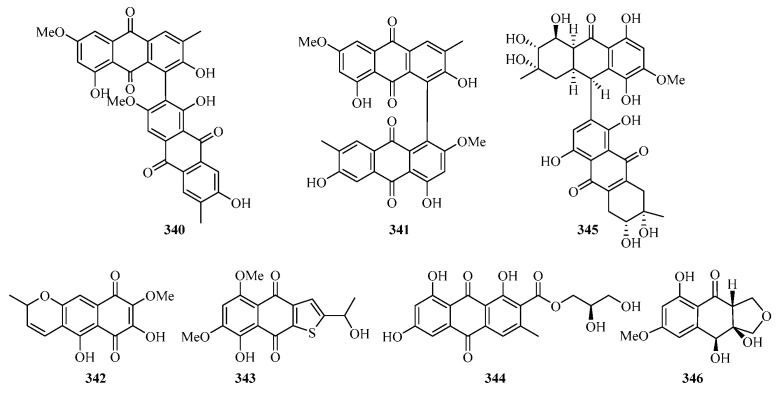
Chemical structures of quinones and xanthones (**324**–**346**).

Two new 1, 4-naphthoquinone derivatives (**324** and **325**) (Figure 21) were obtained from the endophytic fungus *Talaromyces* sp. SK-S009 isolated from the fruit of *Kandelia obovata*. Compound **324** exhibited significantly inhibitory activities against LPS-induced NO production in the murine macrophage cell line, with an IC_50_ value of 3.9 μM [86]. 

Bioassay-guided fractionation of the dichloromethane extract of the fungus *Neofusicoccum austral* SYSU-SKS024 led to the isolation of three new ethylnaphthoquinone derivatives, neofusnaphthoquinone A (**326**), 6-(1-methoxylethy1)-2,7-dimethoxyjuglone (**327**), (3*R*,4*R*)-3-methoxyl-botryosphaerone D (**328**). All of them showed in vitro inhibitory effects against IDO with IC_50_ values ranging from 0.1 to 6.4 μM [87]. 

One new hydroanthraquinone dimer with a rare C-9–C-71 linkage, nigrodiquinone A (**329**), was isolated from a fungus *Nigrospora* sp. obtained from the zoanthid *Palythoa haddoni* collected from the Weizhou Island [88]. A chemical investigation of a marine-derived fungus *Nigrospora* sp., isolated from an unidentified sea anemone, yielded two new hydroanthraquinone analogues, 4a-*epi*-9*α*-methoxydihydrodeoxybostrycin (**330**) and 10-deoxybostrycin (**331**) [89]. 

Five new hydroanthraquinone derivatives, tetrahydroaltersolanols C–F (**332**–**335**) and dihydroaltersolanol A (**336**), and five new alterporriol-type anthranoid dimers, alterporriols N–R (**337**–**341**), were isolated from the culture broth and the mycelia of *Alternaria* sp. ZJ-2008003, a fungus obtained from a *Sarcophyton* sp. soft coral collected from Weizhou Island. Compounds **332** and **340** exhibited antiviral activity against the porcine reproductive and respiratory syndrome virus, with IC_50_ values of 65 and 39 μM, respectively. Compound **339** showed cytotoxic activity against PC-3 and HCT-116 cell lines, with IC_50_ values of 6.4 and 8.6 μM, respectively [90]. 

Leptospyranonaphthazarin A (**342**) was isolated from an endophytic fungus *Leptosphaerulina* sp. SKS032. In the antibiotic assay, compound **342** exhibited antibacterial activity against *Staphylococcus aureus* with an MIC value of 25.0 μg/mL [74]. The mangrove endophytic fungus *Aspergillus terreus* (No. GX7-3B) was cultivated in potato dextrose liquid medium, and one new thiophene compound (**343**) was isolated [91]. DPPH assay of the in-house marine-derived fungi uncovered the EtOAc extract of the cultured fungus *Aspergillus europaeus* WZXY-SX-4-1, which was isolated from the marine sponge *Xestospongia testudinaria*. Chromatographic separation of the bioactive extract resulted in the isolation of one new compound namely (+)1-*O*-demethylvariecolorquinone A (**344**) [68]. A new alterporriol-type anthranoid dimer, alterporriol S (**345**), was isolated from the culture broth of the mangrove fungus, *Alternaria* sp. SK11, from the Weizhou Island [92]. 

A new polyketide, 4*S*,3a*S*,9a*R*-3a,9a-deoxy-3a hydroxy-1-dehydroxyarthrinone (**346**), was isolated and identified from the sponge-derived fungus *Arthrinium* sp. SCSIO 41421. Preliminary bioactivity screening and molecular docking analysis revealed that **346** exhibited obvious enzyme inhibitory activity against AChE, with an inhibitory rate of 84.2% at 50 μg/mL [93]. 

**Figure 22:**
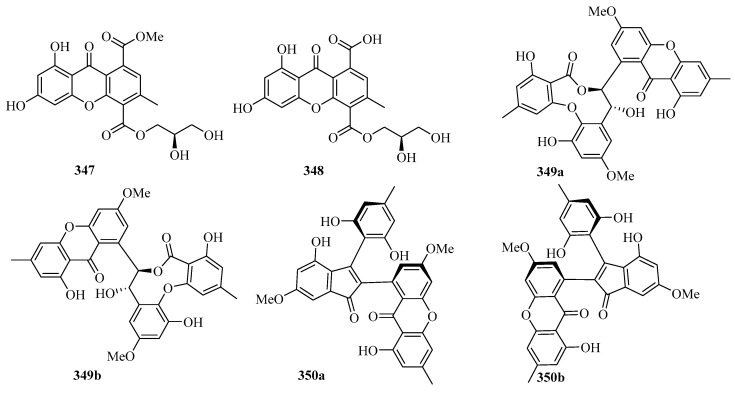

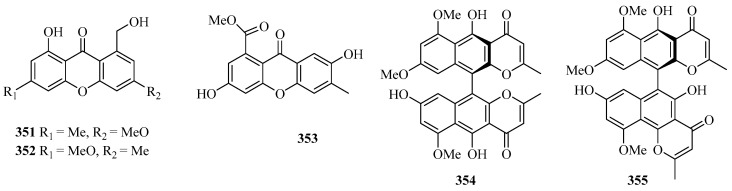
Chemical structures of quinones and xanthones (**347**–**355**).

Chromatographic separation of the bioactive extract resulted in the isolation of two new polyketide derivatives, namely euroxanthones A (**347**) and B (**348**). Moreover, compound **347** (10 μM) (Figure 22) exerted inhibitory activity against NO production in the LPS-induced BV2 cells of 42.2% [68]. 

A pair of novel enantiomeric polyketide dimers, (+)- and (−)-ascomlactone A (**349a** and **349b**) were obtained from a mangrove endophytic fungus *Ascomycota* sp. SK2YWS-L. T. Both of them exhibited significant inhibition effects against *α*-glucosidase with IC_50_ values of 63.7 and 27.9 μM, respectively [94]. 

A pair of novel 2,3-diaryl indone derivatives (+)- and (−)-ascomindone D (**350a** and **350b**) were obtained from the culture of a mangrove endophytic fungus *Ascomycota* sp. SK2YWS-L. In the anti-inflammatory assay, compounds **350a** and **350b** exhibited potential anti-inflammatory effects by inhibiting the production of NO in LPS-induced RAW 246.7 mouse macrophages with IC_50_ values of 17.0 and 17.1 μM, respectively [70]. 

Two new xanthones, 1-hydroxy-8-(hydroxymethyl)-6-methoxy-3-methyl-9*H*-xanthen-9-one (**351**) and 1-hydroxy-8-(hydroxymethyl)-3-methoxy-6-methyl-9*H*-xanthen-9-one (**352**), were isolated from a mangrove endophytic fungus *Phoma* sp. SK3RW1M collected from the Weizhou Island [71]. 

A new xanthone, 2,6-dihydroxy-3-methyl-9-oxoxanthene-8-carboxylic acid methyl ester (**353**) was isolated from the marine fungus *Phomopsis* sp. (No. SK7RN3G1), which exhibited cytotoxicity against HEp-2 and HepG2 cells with IC_50_ values of 8 and 9 ng/mL, respectively [95]. 

Two new dimeric naphtho-*γ*-pyrones, compounds **354** and **355**, were isolated from the EtOAc extract of the fungal strain WZ-4-11 of *Aspergillus carbonarius*. Remarkably, compounds **354** and **355** showed weak antimycobacterial activities against *Mycobacterium tuberculosis* H37Rv, with MIC values of 43.0 and 21.5 μM, respectively [96]. 

#### 2.2.5. Macrolides

**Figure 23:**
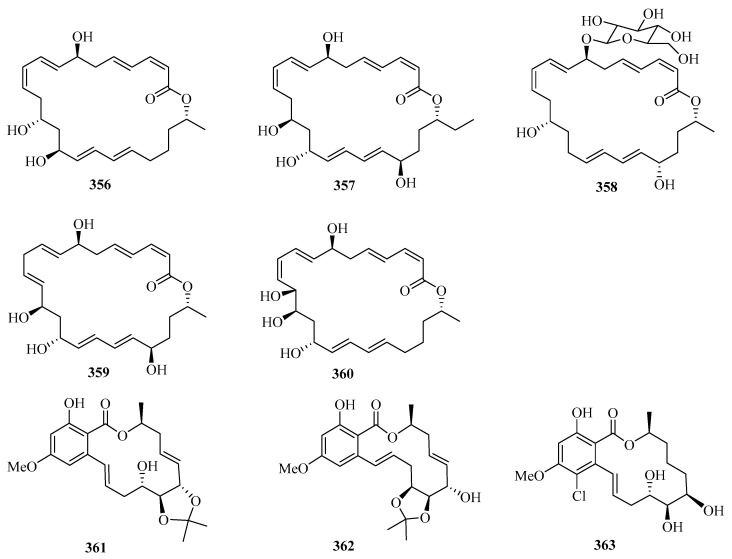

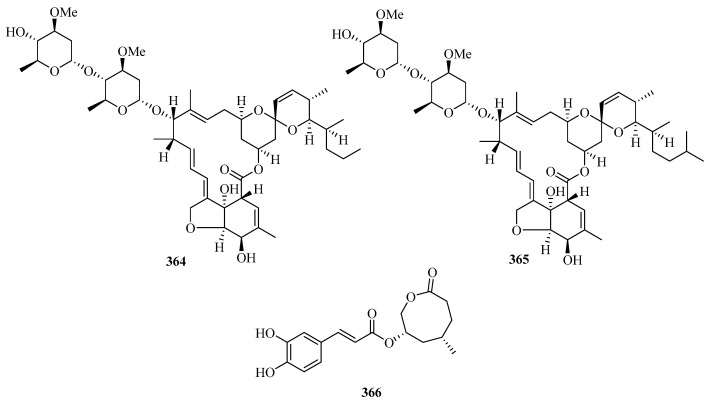
Chemical structures of macrolides (**356**–**366**).

Secondary metabolites of marine-derived *Bacillus siamensis* were isolated and screened for inhibitory activities, which led to the discovery of five new 24-membered macrolactins, bamemacrolactins A–E (**356**–**360**) (Figure 23), with compound **358** being the most potent sugarcane smut fungicide. In addition, compound **358** had inhibitory effects on MAT-1 and MAT-2 of *Sporisorium scitamineum* with EC_50_ values of 0.5 μg/mL and 10.8 μg/mL, respectively [97]. 

Three new 14-membered resorcylic acid lactones, named cochliomycins A–C (**361**–**363**), were isolated from the culture broth of *Cochliobolus lunatus*, a fungus obtained from the gorgonian *Dichotella gemmacea* collected from Weizhou Island. All of these compounds completely inhibited the larval settlement of *Balanus amphitrite* at a concentration of 20.0 μg/mL. Remarkably, compound **361** showed a significant inhibitory effect on larval settlement even at a concentration of 5.0 μg/mL [98]. 

Two new avermectin derivatives, avermectins B1c (**364**) and B1e (**365**), were isolated from a Beibu Gulf gorgonian coral, *Anthogorgia caerulea*. Compounds **364** and **365** showed moderate antifouling activities against the larval settlement of *Balanus amphitrite*, with ED_50_ values of 15.8 and 6.3 μg/mL, respectively [99]. Further chemical investigation of the mangrove fruit *Avicennia marina* led to the isolation of a new caffeic acid derivative, maricaffeolylide A (**366**), which showed antioxidant activity with an EC_50_ value of 24.0 μM [100]. 

#### 2.2.6. Miscellaneous

**Figure 24:**
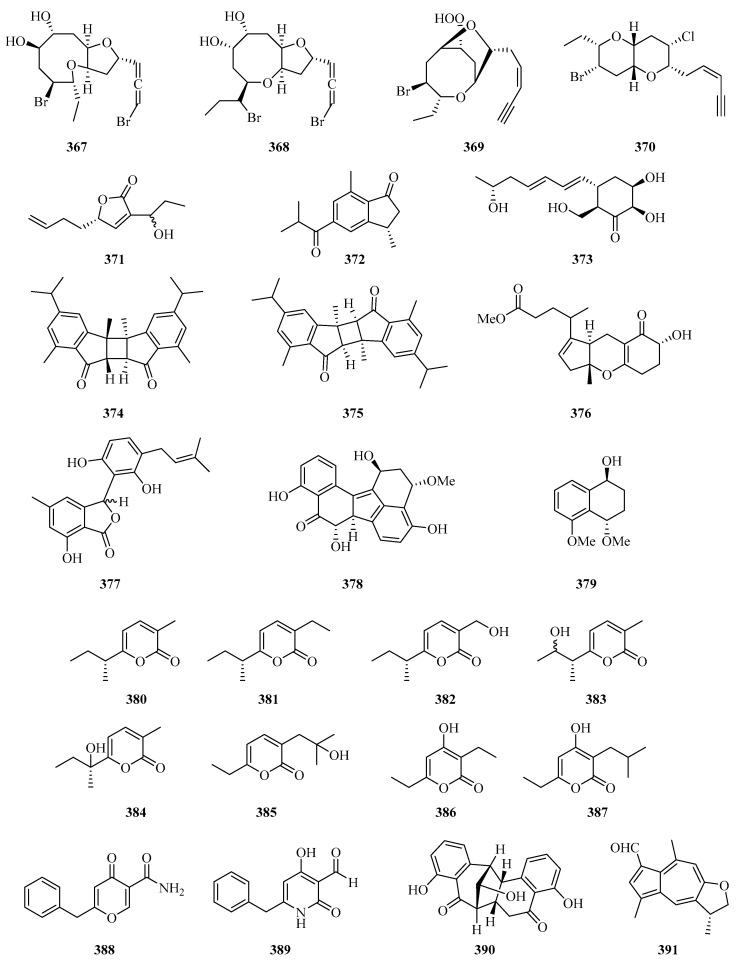

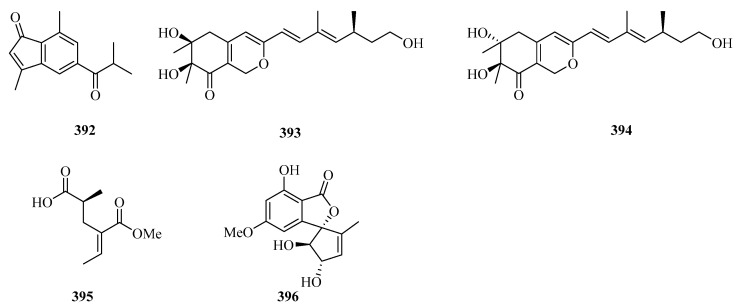
Chemical structures of miscellaneous polyketides (**367**–**396**).

Four new halogenated nonterpenoid C15-acetogenins, laurendecumallene A (**367**), laurendecumallene B (**368**), laurendecumenyne A (**369**), and laurendecumenyne B (**370**) (Figure 24), were isolated and identified from the organic extract of the marine red alga *Laurencia decumbens* [101]. 

One new polyketide, 8-hydroxy-pregaliellalactone B (**371**), was isolated from the mangrove endophytic fungus *Phoma* sp. SYSU-SK-7, which exhibited significant *α*-glucosidase inhibitory activity with an IC_50_ value of 40.8 μM [67]. One new guaiazulene-based compound designated anthogorgienes A (**372**) was isolated from a Chinese gorgonian *Anthogorgia* sp. [50]. One new polyketide was isolated from the mangrove endophytic fungus, *Penicillium* sp. sk14JW2P, named 13-hydroxypalitantin (**373**), which showed AchE inhibitory activity with an IC_50_ value of 12.0 ± 0.3 nM [102]. 

Two uncommon marine gorgonian-derived symmetric dimers, weizhouochrones A (**374**) and B (**375**), with indenone-derived monomers, were isolated from the coral *Anthogorgia ochracea* collected from Weizhou Island [103]. The marine-derived fungus *Alternaria alternata* was isolated from branch samples of Beibu Gulf, Qinzhou City, Guangxi Autonomous Region, China. A chemical study of marine-derived fungus *Alternaria alternata*, led to the isolation of a new isomer compound named tricycloalternarene 18c (**376**) [104]. 

One new prenylated polyketide, ascomfuran C (**377**) was obtained from the culture of a mangrove endophytic fungus *Ascomycota* sp. SK2YWS-L [70]. Two undescribed compounds, guhypoxylonols A (**378**) and B (**379**), were isolated from the mangrove endophytic fungus *Aspergillus* sp. GXNU-Y45. Compound **378** showed inhibitory activity against the production of NO, with IC_50_ values of 14.4 ± 0.1 μM [72]. Eight new *α*-pyrones (**380**–**387**) were isolated from three marine-derived *Nocardiopsis* strains SCSIO 10419, SCSIO 04583, and SCSIO KS107. All of them exhibited moderate growth inhibitory activity against *Micrococcus luteus* and *Bacillus subtilis* [105]. 

Carbonarones A (**388**) and B (**389**) were obtained from the culture of the marine-derived fungus *Aspergillus carbonarius* isolated from the marine sediment collected at Weizhou island of China. Both of them showed moderate cytotoxicities against K562 cells with IC_50_ values of 56.0 and 27.8 μg/mL, respectively [106]. A novel perylenequinone-related compound, alternatone A (**390**), with an unprecedented tricyclo [6.3.1.02,7] dodecane skeleton was isolated from the soft-coral-derived fungus *Alternaria alternata* L3111′ [107]. Two new guaiazulene-based analogues, ochracenoids A (**391**) and B (**392**), were isolated from the gorgonian *Anthogorgia ochracea* collected from the Weizhou Island [108]. 

Two new azaphilones, asperterilones A (**393**) and B (**394**), were isolated from an hdaA mutant. Both of them displayed moderate anti-candida activities against Candida parapsilosis with MIC values of 18.0 and 23.9 μM, respectively (MIC value of amphotericin, 2.2 μM). Additionally, compound **393** exhibited significant cytotoxic activity against human breast cancer cell line MDA-MB-231 [109]. Streptopentanoic acid (**395**), was obtained from the culture of the marine-derived *Streptomyces* sp. MDW-06, which showed DPPH radical scavenging activity with 36.4% at 100 mg/L [110]. One new altenusin derivative (**396**), was isolated from a culture of the endophytic fungus *Alternaria* sp. SK6YW3L, which was isolated from a fresh fruit of the mangrove plant *Sonneratia caseolaris*, collected from Weizhou Island [81].

### 2.3. Nitrogen-Containing Compounds

A total of 58 nitrogen-containing compounds were discovered within the period, consisting of 24 alkaloids, 18 peptides, 16 amides and other miscellaneous ones. In addition, a total of 26 active compounds were found in these nitrogen-containing compounds in the Beibu Gulf, including cytotoxic (15 molecules), anti-microbial (5 molecules), enzyme inhibitory (3 molecules), anti-fouling (2 molecules), and anti-viral activities (1 molecule).

#### 2.3.1. Alkaloids

**Figure 25:**
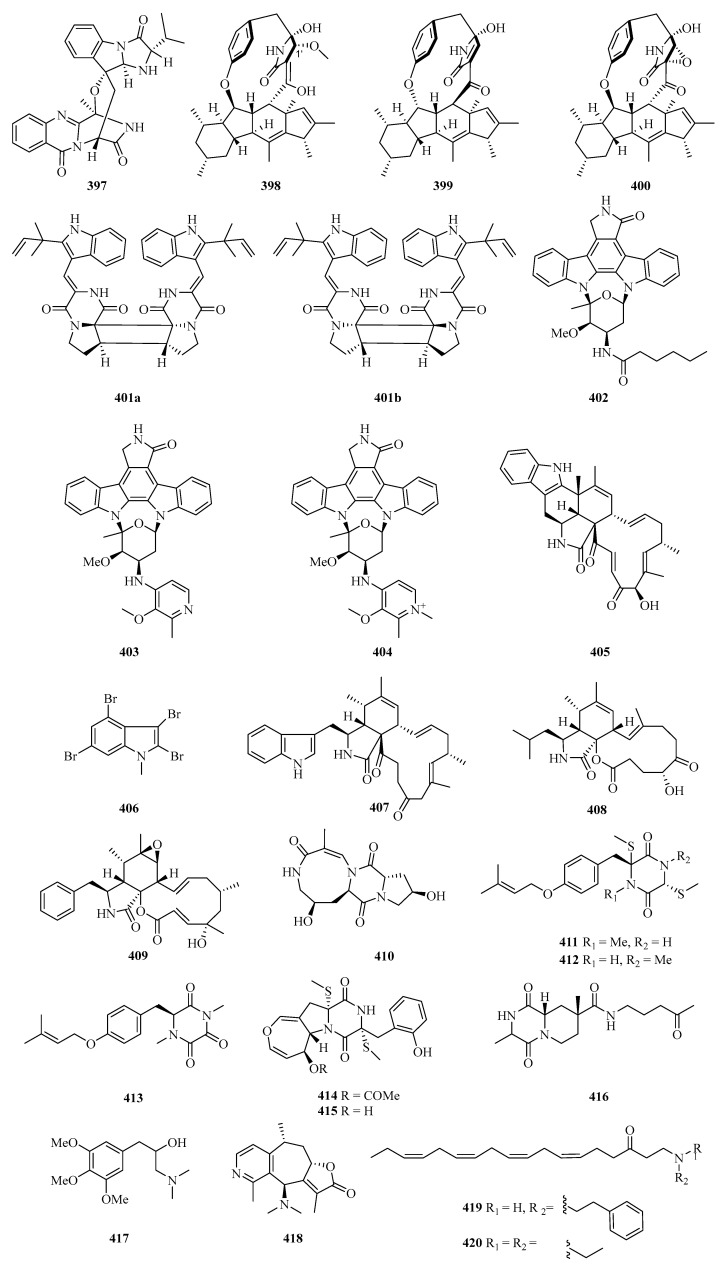
Chemical structures of alkaloids (**397**–**420**).

Fumiquinazoline L (**397**) (Figure 25) is an alkaloid with a heptacyclic skeleton formed via a bridging hemiaminal linkage which was isolated from a gorgonian-derived *Scopulariopsis* sp. fungus [111]. Three new 12- or 13-membered-ring macrocyclic alkaloids, named ascomylactams A–C (**398**–**400**), were isolated from the mangrove endophytic fungus *Didymella* sp. CYSK-4. Compounds **398** and **400** showed moderate cytotoxicities against MDA-MB-435, MDA-MB-231, SNB19, HCT116, NCI-H460, and PC-3 human cancer cell lines, with IC_50_ values in the range of 4.2–7.8 μM [112]. 

A pair of enantiomeric indole diketopiperazine alkaloid dimers, (−)- and (+)-asperginulin A (**401a** and **401b**), with an unprecedented 6/5/4/5/6 pentacyclic skeleton were isolated from the mangrove endophytic fungus *Aspergillus* sp. SK-28. Interestingly, compound **401b** exhibited antifouling activity against the barnacle *Balanus reticulatus*. Moreover, it also inhibited the settlement of the larvae with an adhesive rate of 48.4 ± 0.6% at 10 μg/cm^2^ [113]. 

Under the guidance of global natural product social molecular networking, three new indolocarbazoles named streptocarbazoles F–H (**402**–**404**), were isolated from the marine-derived *Streptomyces* sp. OUCMDZ5380. All of them showed selective antiproliferation of the acute myeloid leukemia cell line MV4-11 with the IC_50_ values of 0.8, 0.6, and 1.9 μM, respectively [114]. 

A novel chaetoglobosin named penochalasin I (**405**) with an unprecedented six-cyclic 6/5/6/5/6/13 fused ring system, and another new chaetoglobosin named penochalasin J (**406**), were isolated from the culture of *Penicillium chrysogenum* V11. Surprisingly, compound **406** greatly inhibited *Colletotrichum gloeosporioide* (MIC = 25.1 μM), showing an antifungal activity higher than that of carbendazim. In addition, compound **405** exhibited marked cytotoxicity against MDA-MB-435 and SGC-7901 cells, with the IC_50_ values of 7.6 and 7.3 μM, respectively [115]. 

One new polybromoindole, 2,3,4,6-tetrabromo-1-methyl-1*H*-indole (**407**), was isolated and identified from the marine red alga *Laurencia decumbens* [30]. Two new cytochalasins, aspochalasin A1 (**408**) and cytochalasin Z24 (**409**), were isolated from the fermentation broth of *Aspergillus elegans* ZJ-2008010, a fungus obtained from a soft coral *Sarcophyton* sp., collected from south Weizhou Island [116]. 

Chemical investigation on the gorgonian *Menella kanisa* collected from Beibu Gulf led to the isolation of a new diketopiperazine, named menazepine A (**410**) [117]. Another three new diketopiperazine derivatives, saroclazines A–C (**411**–**413**), were isolated from mangrove-derived fungi *Sarocladium kiliense* HDN11-84. The cytotoxic activity of new compounds **411**–**413** was tested against HeLa cell lines, among which compound **412** showed cytotoxicity with an IC_50_ value of 4.2 μM [118]. 

Two new thiodiketopiperazines, emestrins L (**414**) and M (**415**), were obtained from the marine-derived fungus *Aspergillus terreus* RA2905. Particularly, compound **415** displayed antibacterial activity against *Pseudomonas aeruginosa* ATCC 27853 with an MIC value of 64 μg/mL [119]. Chemical investigation of *Nocardiopsis alba,* isolated from *Anthogorgia caerulea,* led to the isolation of a new diketopiperazine, nocarazepine A (**416**) [120]. 

A new alkaloid (**417**) was isolated from the hypocotyl of a mangrove plant, *Bruguiera gymnorrhiza,* and purified with repeated column chromatography on silica and HPLC. It showed anti-Hepatitis B virus activity against HbsAg and HbeAg with IC_50_ values of 4.4 and 4.9 μM, and TI values of 2.7 and 2.4, respectively [121]. Echinoflorine (**418**), a new dimethylamino-substituted guaipyridine alkaloid with a novel *γ*-lactone-cyclohepta [c]pyridine-fused skeleton, was isolated from the gorgonian *Echinogorgia flora,* collected in Weizhou Island [17]. Two new uncommon polyunsaturated amino ketones, (6*Z*,9*Z*,12*Z*,15*Z*)-1-[(2-phenylethyl) amino] octadeca-6,9,12,15-tetraen-3-one (**419**) and (6*Z*,9*Z*,12*Z*,15*Z*)-1-(diethylamino) octadeca-6,9,12,15-tetraen-3-one (**420**), were isolated from the Guangxi Autonomous Region sponge *Haliclona* sp. [122]. 

#### 2.3.2. Peptides

**Figure 26:**
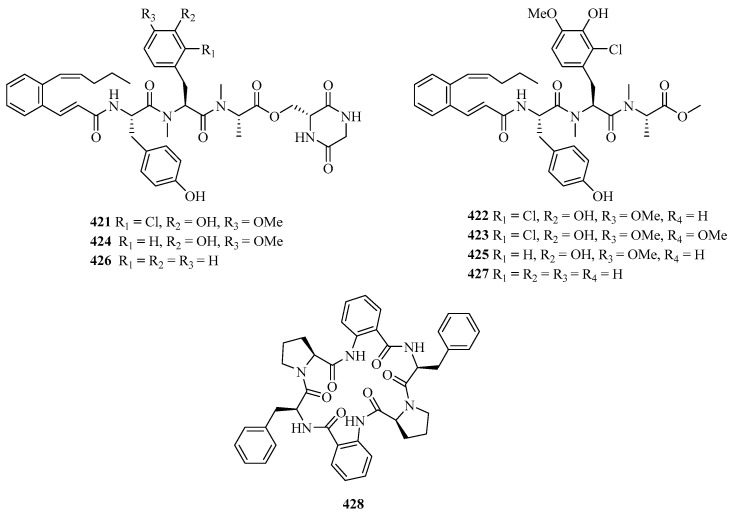
Chemical structures of peptides (**421**–**428**).

Pepticinnamins G–I (**421**–**423**) (Figure 26) were obtained from a marine *Streptomyces* sp. PKU-MA01144 and pepticinnamins J–M (**424**–**427**) and came from several mutants. These new compounds contain different *N*-methyl-*L*-alanine and *L*-tyrosine residues to pepticinnamin E [123]. 

A new centrosymmetric cyclohexapeptide, aspersymmetide A (**428**), was isolated from the fungus *Aspergillus versicolor* isolated from a gorgonian coral *Carijoa* sp., collected from Weizhou Island. Remarkably, it displayed weak cytotoxicity against NCI-H292 and A431 cells with an inhibition ratio of 53.8% and 63.6% at a concentration of 10 μM (adriamycin, 1 μM, 93.4% and 91.0%), respectively [124].

**Figure 27:**
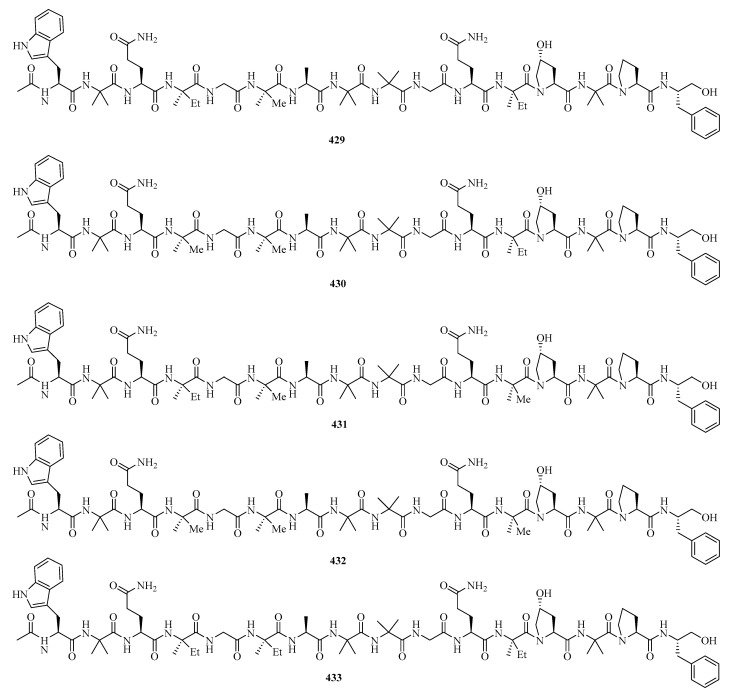

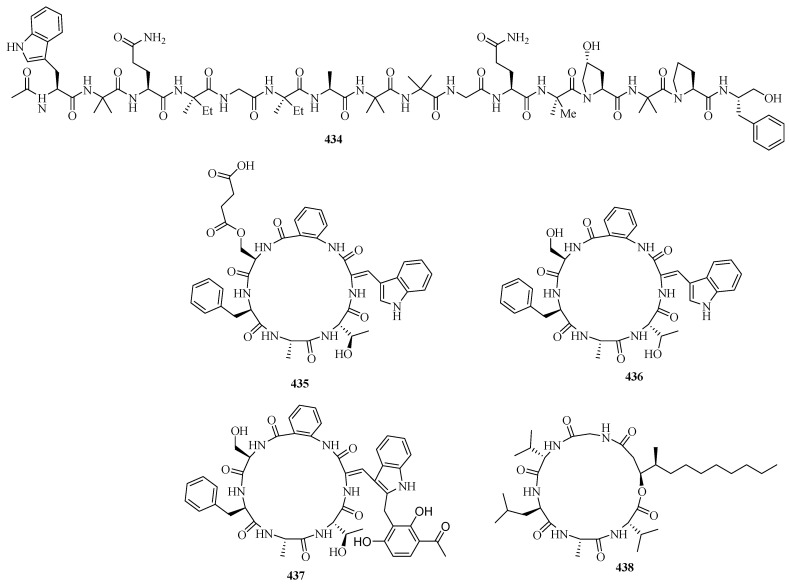
Chemical structures of peptides (**429**–**438**).

Six new 16-residue peptaibols, acremopeptaibols A–F (**429**–**434**) (Figure 27), were isolated from the cultures of the sponge-associated fungus *Acremonium* sp. IMB18-086 was grown in the presence of the autoclaved bacterium *Pseudomonas aeruginosa* on solid rice medium. Compounds **429** and **433** exhibited significant antimicrobial activities against methicillin-resistant *Staphylococcus aureus*, *Bacillus subtilis*, and *Candida albicans*, with MIC values in the range of 16.0–64.0 μg/mL [9]. 

Three novel cyclic hexapeptides, sclerotides C–E (**435**–**437**), and a new lipodepsipeptide, scopularide I (**438**), were isolated from fermented rice cultures of a soft coral-derived fungus *Aspergillus sclerotiorum* SCSIO 41031. Particularly, compound **438** exhibited acetylcholinesterase inhibitory activity with an IC_50_ value of 15.6 μM and weak cytotoxicity against the human nasopharyngeal carcinoma cell line HONE-EBV with an IC_50_ value of 10.1 μM [125]. 

#### 2.3.3. Amides and Miscellaneous

**Figure 28:**
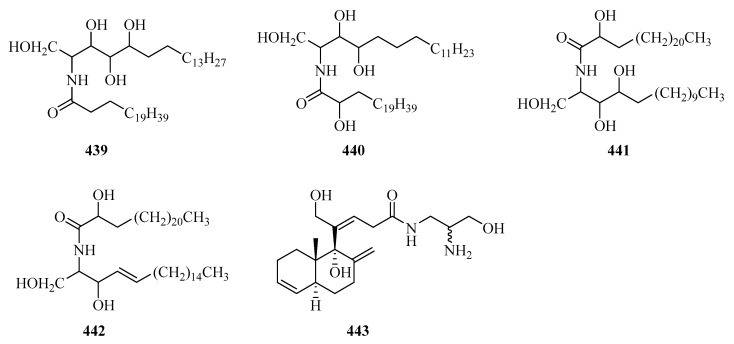
Chemical structures of amides and miscellaneous (**439**–**443**).

Two new ceramides (**439** and **440**) (Figure 28) were isolated from the sponge *Sigmadocia* sp. collected from Weizhou Island [126]. Meanwhile, another two new ceramides, named spongiamines A (**441**) and B (**442**), were also isolated from the sponge *Spongia* sp., collected from Weizhou Island [127]. Kanaphthalene (**443**), was obtained from *Menella kanisa*, which showed significant antifouling activity against the larval settlement of *Balanus amphitrite*, with an EC_50_ value of 5.2 and a LC_50_ value of 55.3 μg/mL [128]. 

**Figure 29:**
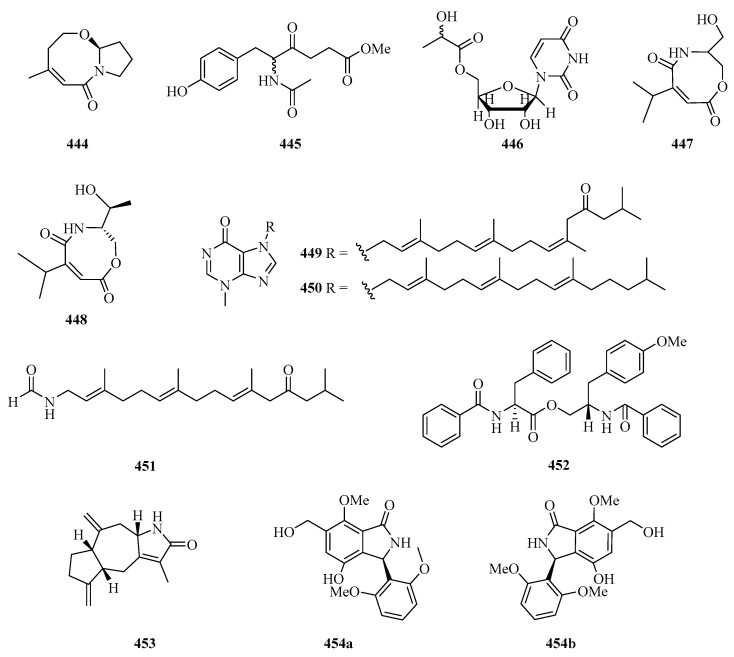
Chemical structures of amides and miscellaneous (**444**–**454**).

One new bicyclic lactam, cladosporilactam A (**444**) (Figure 29), was isolated from a gorgonian-derived *Cladosporium* sp. fungus collected from Weizhou Island, which exhibited promising cytotoxic activity against the cervical cancer HeLa cell line with an IC_50_ value of 0.8 μM [129]. One new 6-phenylhexanoic acid derivative, named 5-acetamido-6(4-hydroxyphenyl)-4-oxohexanoate (**445**), and one new derivative of uridine, named uridine-50a-hydroxypropanoate (**446**), were purified from the extracts of fungus Z18-17 (*Nigrospora* sp.) isolated from *Scyphiphora hydrophyllacea*, a tree from the intertidal zone of Shankou, Guangxi Autonomous Region, China [130]. 

New carboxamides, (±)-vochysiamide C (**447**) and (+)-vochysiamide B (**448**), were isolated and identified from the sponge-derived fungus *Arthrinium* sp. SCSIO 41421. Preliminary bioactivity screening and molecular docking analysis revealed that compound **448** exhibited obvious enzyme inhibitory activity against AChE, with an inhibitory rate of 79.4% at 50 μg/mL [93]. 

Three achiral tetraprenylated alkaloids, named malonganenones I–K (**449**–**451**), were isolated from the gorgonian *Euplexaura robusta* collected from Weizhou Island. Among them, compound **449** showed moderate cytotoxicity against K562 and HeLa tumor cell lines with IC_50_ values of 8.7 and 10.8 μM, respectively [131]. One new phenylalanine derivative, 4′-OMe-asperphenamate (**452**), was isolated from the fermentation broth of *Aspergillus elegans* ZJ-2008010, a fungus obtained from a soft coral *Sarcophyton* sp. collected from Weizhou Island [116]. Taenialactam C (**453**), was isolated from wild *Phaeocystis globose*, which showed significant lethality on the brine shrimp *Artemia salina* and the juvenile *Epinephelus akaara* fish, with a LC_50_ value of 3.1 μg/mL [132]. 

A pair of 3-arylisoindolinone enantiomers, (+)-asperglactam A (**454a**) and (−)-asperglactam A (**454b**) were obtained from the mangrove endophytic fungus *Aspergillus versicolor* SYSU-SKS025. The enantiomers of **454** showed moderate inhibitory activity against *α*-glucosidase with an IC_50_ value of 50.5 μM [75]. 

### 2.4. Glucosides 

**Figure 30:**
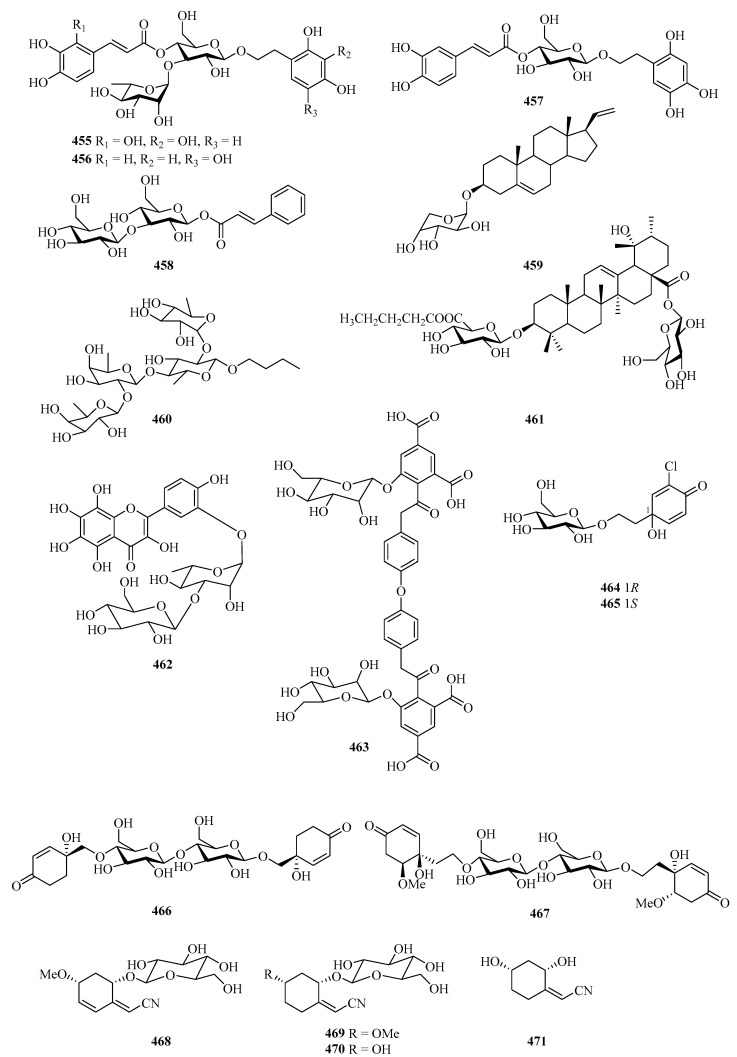

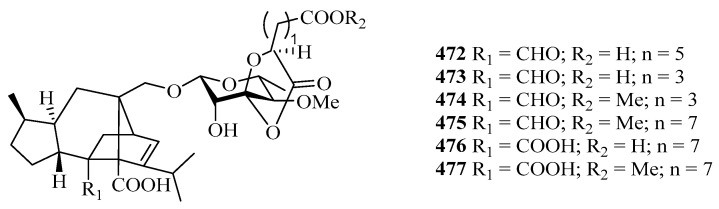
Chemical structures of glucosides (**455**–**477**).

A chemical investigation of the fruit of the mangrove plant, *Avicennia marina*, afforded three new phenylethyl glycosides, marinoids J–L (**455**–**457**), and a new cinnamoyl glycoside, marinoid M (**458**) (Figure 30). The antioxidant activity of the isolates was evaluated using the cellular antioxidant assay, and compounds **455**–**458** showed antioxidant activities, with EC_50_ values ranging from 23.0 ± 0.7 μM to 247.8 ± 2.5 μM [133]. 

One new glycoside (**459**) was isolated from *Cladiella krempfi*, collected from Weizhou Island [134]. A new glycoside compound (**460**) was isolated from the starfish *Asteria amurensis* Lutken. The effects of compound **460** on UMR106 cell proliferation were screened by MTT assay and it (0.01–100 μM) significantly promotes osteoblastic proliferation [135]. 

One new triterpenoid, named saponin (**461**), was isolated from the fruits of *Avicennia marina* in Guangxi Autonomous Region, China, which showed cytotoxicity against GSC3# and GSC-18# with IC_50_ values 12.2 and 5.5 μg/mL, respectively [136]. Further chemical investigation of the plant of *Bruguiera gymnorrhiza* led to the isolation of a new 8-hydroxyquercetagetin glycoside, rugymnoside A (**462**). It showed antioxidant activity with an EC_50_ value of 11.8 ± 0.8 μM [137]. 

The chemical investigation of the fruits of a mangrove *Sonneratia apetala* collected from the Beibu Gulf led to the isolation of one new compound, sonneradon D (**463**) [79]. Four new jacaranone analogs, marinoids F–I (**464**–**467**), were isolated from the fruits of a Beibu Gulf mangrove *Avicennia marina*. Moreover, the antioxidant activity of the isolates was evaluated using a cellular antioxidant assay, and compound **467** showed moderate antioxidant activity, with an EC_50_ value of 26 μM [138]. 

Four new cyclohexylideneacetonitrile derivatives, named menisdaurins B–E (**468**–**471**), were isolated from the hypocotyl of a mangrove plant, *Bruguiera gymnorrhiza*. All of them showed anti-HBV activities, with EC_50_ values ranging from 5.1 ± 0.2 to 87.7 ± 5.8 μg/mL [139]. Six new sordarin tetracyclic diterpene glycosides, moriniafungins B–G (**472**–**477**), were isolated from the zoanthid-derived fungus *Curvularia hawaiiensis* TA26-15. Among them, compound **475** showed antifungal activity against *Candida albicans* ATCC10231 with an MIC value of 2.9 μM [140].

## 3. Discussion and Conclusions

**Figure 31:**
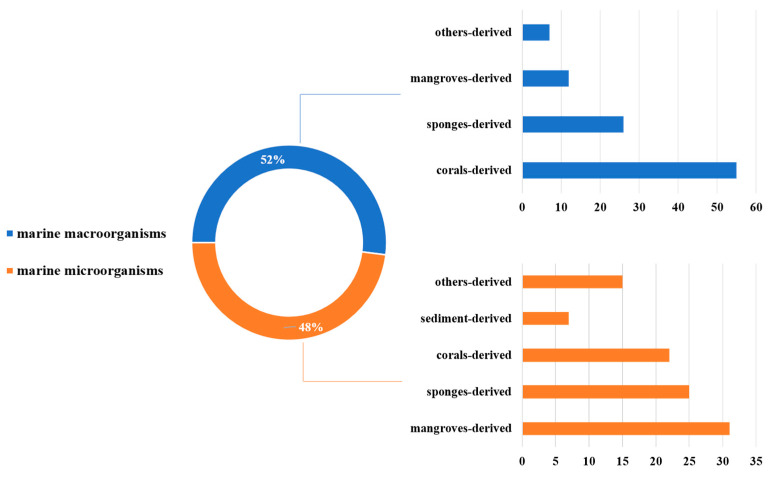
Distribution of marine habitats producing MNPs in the Beibu Gulf from November 2003 to September 2022.

**Figure 32:**
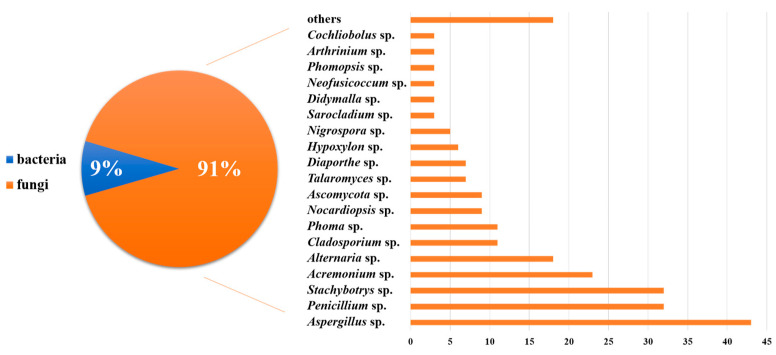
The MNPs from the Beibu Gulf from November 2003 to September 2022 were divided by sources.

MNPs have contributed significantly to modern drug development. We have summarized the sources, structural diversity, and biological activity of 477 newly reported MNPs from the Beibu Gulf region, according to a survey of the literature published from November 2003 to September 2022. This gives us an opportunity to count the MNPs from the Beibu Gulf from the above-mentioned point of view. These reviewed MNPs from Beibu Gulf were found in marine microorganisms (52%) and macroorganisms (48%). In addition, the distribution of these MNPs from macroorganisms and microorganisms was also analyzed. MNPs from macroorganisms were mainly distributed in corals (55%), sponges (26%), and mangroves (12%). However, the top three hosts of microbial-derived MNPs were mangroves (31%), sponges (25%), and corals (22%) (Figure 31). Notably, these MNPs from microorganisms were isolated from 22 genera of fungi (91%) and 6 strains of bacteria (9%). Meanwhile, *Aspergillus* (17%), *Penicillium* (13%), and *Stachybotrys* (13%) were the top three fungal genera from the Beibu Gulf for producing new MNPs (Figure 32).

**Figure 33:**
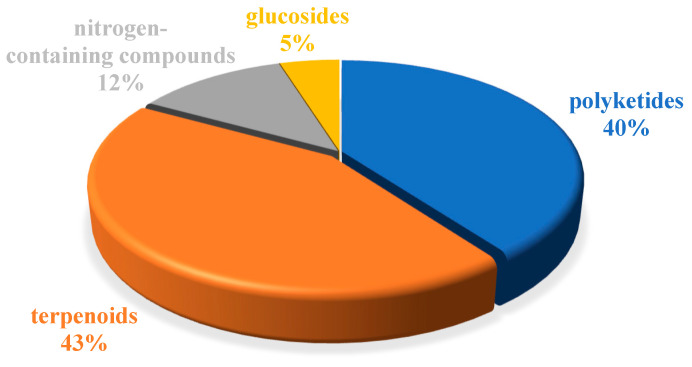
Structural diversity distribution of MNPs in the Beibu Gulf from November 2003 to September 2022.

**Figure 34:**
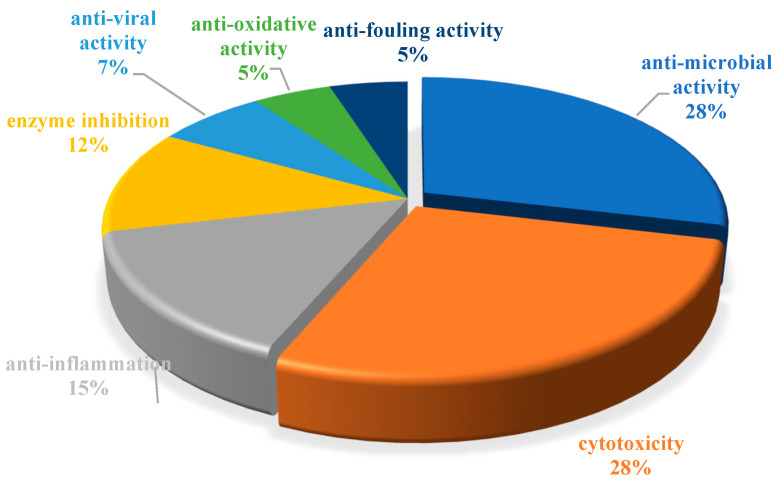
Biological activity distribution of MNPs in the Beibu Gulf from November 2003 to September 2022.

**Figure 35:**
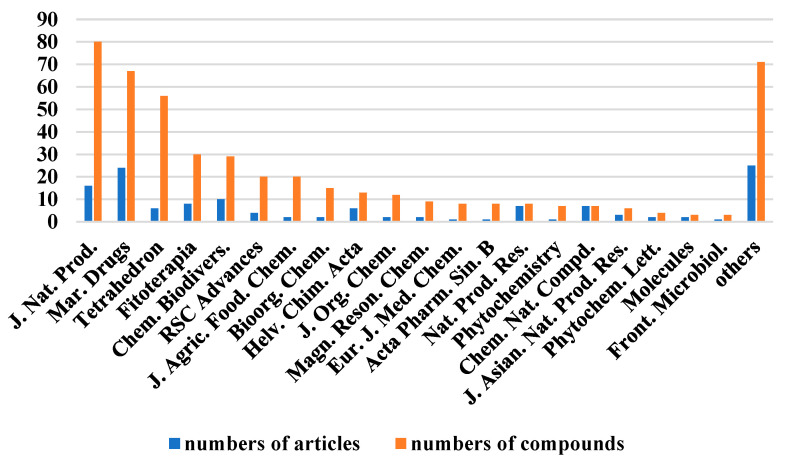
The top 20 periodicals of the number of related articles and compounds of MNPs in the Beibu Gulf published from November 2003 to September 2022.

**Figure 36:**
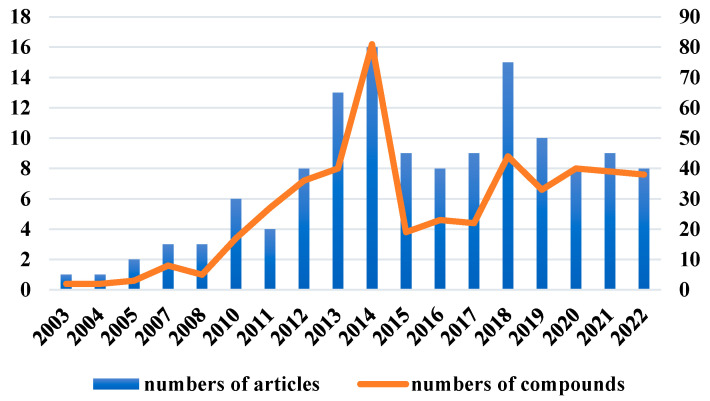
Number of articles and compounds related to MNPs in the Beibu Gulf in each year from November 2003 to September 2022.

**Figure 37:**
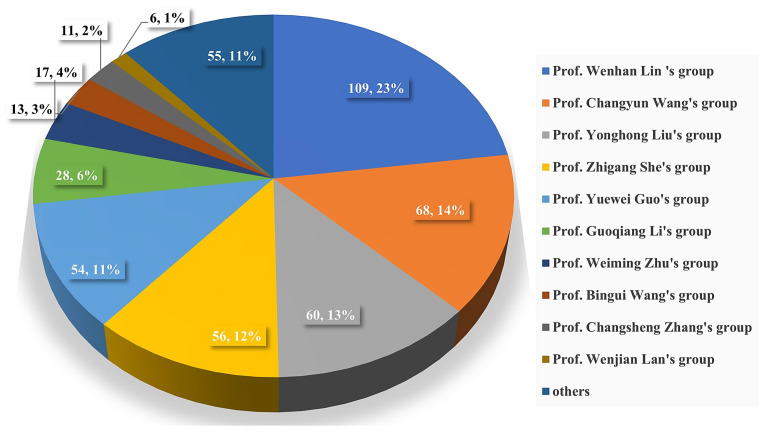
The top 10 known research groups in China for discovering new MNPs from the Beibu Gulf during 2003–2022.

The chemical structures of the derived MNPs were divided into four categories as shown in Figure 33. Remarkably, terpenoids (43%), polyketides (40%), and nitrogen-containing compounds (12%) ranked as the top three structural types, followed by glucosides (5%). Strikingly, nearly 39% of them (184 of 477 compounds) were discovered with a broad spectrum of bioactivities, including anti-microbial (28%), cytotoxic (28%), anti-inflammatory (15%), enzyme inhibitory (12%), anti-viral (7%), anti-oxidative (5%), and anti-fouling activities (5%) (Figure 34). In addition, a total of 133 articles involving 477 newly reported MNPs have been published in 31 kinds of related international journals in the past twenty years (Figure 35). Interestingly, among them, 67 new MNPs (14%) in 24 articles (18%) were published in *Marine Drugs*, which was the most populous journal among these references. This review also counted the number of articles and compounds from new MNPs in the Beibu Gulf published annually from November 2003 to September 2022 (Figure 36). The statistical results showed that the number of new MNPs originating from the Beibu Gulf increased year by year from 2003 to 2014, with explosive growth from 2011 to 2014. After the rate of discovery of new compounds fell back in 2015, new compounds were discovered at a steady rate every year. Finally, we counted the top 10 research groups in China that reported the largest amount of new MNPs in the Beibu Gulf from November 2003 to September 2022, among which Prof. Wenhan Lin’s group (23%), Prof. Changyun Wang’s group (14%), and Prof. Yonghong Liu’s group (our team) (13%) are in the top three research groups, and 109, 68, and 60 NMPs were discovered by them, respectively (Figure 37). It is worth noting that marine habitats have become the main source of halogenated compounds because the concentrations of bromine and chloride ions in sea water are higher than those in the terrestrial environment [141]. According to the literature survey from November 2003 to September 2022, 58 (12%) halogenated natural products were found in the 477 MNPs newly reported from the Beibu Gulf, which indicates the Beibu Gulf is one of the important areas for producing halogenated MNPs.

Based on the foregoing discussion, over the past 20 years (from November 2003 to September 2022), 477 new natural products were reported in 133 papers. Although the research on secondary metabolites from Beibu Gulf has only been going for 20 years, more attention has been paid to it in recent years. In this review, it seems that fungi are the focus of biological prospects for bioactive metabolites of microorganisms in the Beibu Gulf. Among bacteria, actinomycetes seem to be studied more deeply in natural product research, and they have shown the potential to become biological resources with novel structures and good biological activities. In terms of structural category, polyketides occupy the largest number and show a wide range of biological activities, such as anti-tumor, antibacterial, and anti-inflammatory activities. So far, many well-known research groups in China, represented by Prof. Wenhan Lin, Prof. Changyun Wang, and Prof. Yonghong Liu, have reported a large number of MNPs with novel structures and promising activity. The significant biological activity of these MNPs from the Beibu Gulf is dominated by cytotoxicity (28%) and anti-microbial activity (28%). By exploring untapped novel species of marine organisms and by proposing newer biological targets, it would enhance the possibility for discovering novel lead compounds with potential therapeutic applications. Moreover, careful and innovative techniques for MNPs isolation are required for the identification of new structures and activities, including unstable intermediates. In addition to exploring potential new natural biological resources, the application of drug discovery-related techniques, such as gene mining and combinatorial biosynthesis, will improve the structural and biological diversity of MNPs. A large number of the Beibu Gulf marine macro- and microorganisms are still underexplored, and they may provide inspiration for many chemical entities. Therefore, the natural product resources in the Beibu Gulf are still a virgin land to be urgently developed. Reasonable and green application research will contribute more to drug discovery.

## Abbreviations

MNPs	Marine natural products
MIC	Minimum Inhibiting Concentration
MBC	Minimum Bactericidal Concentration
NF-κB	Nuclear Factor Kappa B
LPS	Lipopolysaccharide
TNBC	Triple-Negative Breast Cancer
